# Closing the gap: Oxford Nanopore Technologies R10 sequencing allows comparable results to Illumina sequencing for SNP-based outbreak investigation of bacterial pathogens

**DOI:** 10.1128/jcm.01576-23

**Published:** 2024-03-05

**Authors:** Bert Bogaerts, An Van den Bossche, Bavo Verhaegen, Laurence Delbrassinne, Wesley Mattheus, Stéphanie Nouws, Maxime Godfroid, Stefan Hoffman, Nancy H. C. Roosens, Sigrid C. J. De Keersmaecker, Kevin Vanneste

**Affiliations:** 1 Transversal activities in Applied Genomics, Sciensano, Brussels, Belgium; 2 Bacterial Diseases, Sciensano, Brussels, Belgium; 3 Foodborne Pathogens, Sciensano, Brussels, Belgium; Cleveland Clinic, Cleveland, Ohio, USA

**Keywords:** outbreak, nanopore sequencing, illumina, public health, whole-genome sequencing

## Abstract

Whole-genome sequencing has become the method of choice for bacterial outbreak investigation, with most clinical and public health laboratories currently routinely using short-read Illumina sequencing. Recently, long-read Oxford Nanopore Technologies (ONT) sequencing has gained prominence and may offer advantages over short-read sequencing, particularly with the recent introduction of the R10 chemistry, which promises much lower error rates than the R9 chemistry. However, limited information is available on its performance for bacterial single-nucleotide polymorphism (SNP)-based outbreak investigation. We present an open-source workflow, Prokaryotic Awesome variant Calling Utility (PACU) (https://github.com/BioinformaticsPlatformWIV-ISP/PACU), for constructing SNP phylogenies using Illumina and/or ONT R9/R10 sequencing data. The workflow was evaluated using outbreak data sets of Shiga toxin-producing *Escherichia coli* and *Listeria monocytogenes* by comparing ONT R9 and R10 with Illumina data. The performance of each sequencing technology was evaluated not only separately but also by integrating samples sequenced by different technologies/chemistries into the same phylogenomic analysis. Additionally, the minimum sequencing time required to obtain accurate phylogenetic results using nanopore sequencing was evaluated. PACU allowed accurate identification of outbreak clusters for both species using all technologies/chemistries, but ONT R9 results deviated slightly more from the Illumina results. ONT R10 results showed trends very similar to Illumina, and we found that integrating data sets sequenced by either Illumina or ONT R10 for different isolates into the same analysis produced stable and highly accurate phylogenomic results. The resulting phylogenies for these two outbreaks stabilized after ~20 hours of sequencing for ONT R9 and ~8 hours for ONT R10. This study provides a proof of concept for using ONT R10, either in isolation or in combination with Illumina, for rapid and accurate bacterial SNP-based outbreak investigation.

## INTRODUCTION

Whole-genome sequencing (WGS) has become established as a powerful method for investigating outbreaks of bacterial pathogens. It has been proven highly successful in elucidating the transmission dynamics and evolutionary relationships among isolates, thereby rapidly resolving bacterial outbreaks and aiding in the implementation of effective control measures (1, 2). Short-read Illumina sequencing is currently considered the gold standard for WGS, in part because of its low error rate, which allows a resolution down to the single nucleotide, and its cost-effectiveness through multiplexing when sequencing many samples (3). In recent years, long-read Oxford Nanopore Technologies (ONT) sequencing has emerged as a promising alternative. ONT sequencing generates longer reads that can cover repetitive regions, resulting in a larger fraction of the genome that can be covered. Additionally, ONT offers small sequencing devices with short turnaround times and a lower initial investment, enabling rapid data generation that is also cost-effective when only few samples need to be sequenced (4). Due to recent improvements such as reduced error rates (although still higher than Illumina), increased throughput, and the refinement of data analysis and wet-lab protocols, public health and clinical laboratories are experimenting with this technology to evaluate its added value for integration into their routine activities (4–8).

WGS can provide a much higher resolution than conventional methods such as pulse-field gel electrophoresis or multi-locus sequence typing (MLST) for investigating bacterial outbreaks (2, 9, 10). WGS enables core genome multi-locus sequence typing (cgMLST), which has become the standard for relatedness and outbreak investigation due to its improved scalability, generally high inter-laboratory reproducibility, and independence from a defined reference strain (11). Moreover, WGS has the added advantage of providing a complete characterization of bacterial isolates during outbreak investigations, including the detection of antimicrobial resistance and virulence genes, mobile genetic elements, and other genomic features that may affect the phenotypic properties of the strain (2). However, cgMLST still uses only a fraction of the genome to delineate strains. Single-nucleotide polymorphism (SNP)-based phylogenetics provides the highest possible resolution, down to the single nucleotide, to characterize relationships between isolates across their entire genomes, which is particularly valuable for closely related strains. Despite the lack of standardization, it has therefore become an established method in bacterial outbreak investigation, for example, to understand the spread and transmission of a pathogen or to identify the exact source of foodborne outbreaks (12). Numerous variant callers and SNP phylogeny workflows are therefore available (13–15), and their performance has been documented in several case studies (13, 16, 17). Although a consensus methodology is lacking, most commonly used variant callers accurately identify SNPs in Illumina data (15). Due to the relatively recent advent and rapid evolution of ONT sequencing, corresponding SNP-based phylogeny workflows are much less established, and there is less information available on their performance in delineating outbreak strains, but several recent case studies have highlighted the potential of ONT sequencing for accurate SNP identification and precise taxonomic placement of bacterial isolates (4, 6, 8, 18, 19). Additionally, while the number of case studies demonstrating the potential of ONT sequencing for SNP-based analysis is high, the number of studies integrating samples sequenced by either ONT or Illumina into the same phylogenetic analysis is still limited. This is nevertheless relevant for outbreak analysis, as it is a prerequisite for constructing phylogenies with data generated by laboratories using different sequencing technologies and for comparison with historical samples. Several tools and workflows that can process both ONT data and Illumina data are available (18, 20, 21), but little information is available on their performance when handling both types of data.

In 2022, ONT released the new R10 sequencing chemistry, which is expected to increase read accuracy compared to the previous, commonly used R9 chemistry, which will be phased out in 2024. Additionally, “duplex base calling” was introduced, which is claimed to provide Q30 (i.e., 99.9% accuracy) single-molecule reads, corresponding to an error rate in the same range as Illumina sequencing. Duplex base calling incorporates the base calling information from both strands of double-stranded DNA molecules to improve read accuracy in a second round of base calling. In theory, the increased accuracy should be beneficial for SNP calling, but the performance has not yet been systematically evaluated, and case studies using the new R10 chemistry specifically for SNP-based bacterial outbreak resolution remain scarce or even non-existent at the time of writing. Recent studies have shown improved performance of the R10 chemistry over the R9 chemistry for cgMLST-based analysis and genome reconstruction, raising the question of whether the performance of SNP-based phylogenetic analysis would also benefit from the improved read quality (22, 23).

One advantage of ONT sequencing is the ability to perform live base calling, meaning that data can be analyzed as they are being generated, enabling a potentially faster response in crisis situations (22). Several case studies have used ONT sequencing to optimize the response time for various WGS-based applications, including the identification of closely related *Neisseria gonorrhoeae* (6), confirmation of a clinical methicillin-resistant *Staphylococcus aureus* outbreak (4), and cgMLST-based real-time surveillance of various bacterial pathogens (22). The required sequencing time depends on many factors, including the type of assay, sequencing chemistry, and target species. However, in all cases, ONT sequencing could drastically reduce the overall turnaround time for these applications. However, information on the time or throughput required for the new R10 chemistry to provide reliable information for delineating outbreak clusters through SNP-based approaches is still absent.

We present the Prokaryotic Awesome variant Calling Utility (PACU) workflow for resolving bacterial outbreaks using a SNP-based approach, with built-in variant filters that account for the higher error rate of ONT data. We assessed the performance of the workflow using either R9 or R10 compared to the Illumina data from historical outbreaks of Shiga toxin-producing *Escherichia coli* (STEC) and *Listeria monocytogenes* as case studies. We also evaluated how these technologies perform when used within the same phylogenomic analysis, since outbreak investigations are often conducted as collaborations between multiple laboratories that may use different sequencing technologies. Finally, we evaluated the performance in function of sequencing time to determine the minimum time to obtain accurate results when using R9 or R10, as a rapid response is critical in outbreak scenarios to minimize the negative impact.

## MATERIALS AND METHODS

### Collection of bacterial isolates

**Fig 1:**
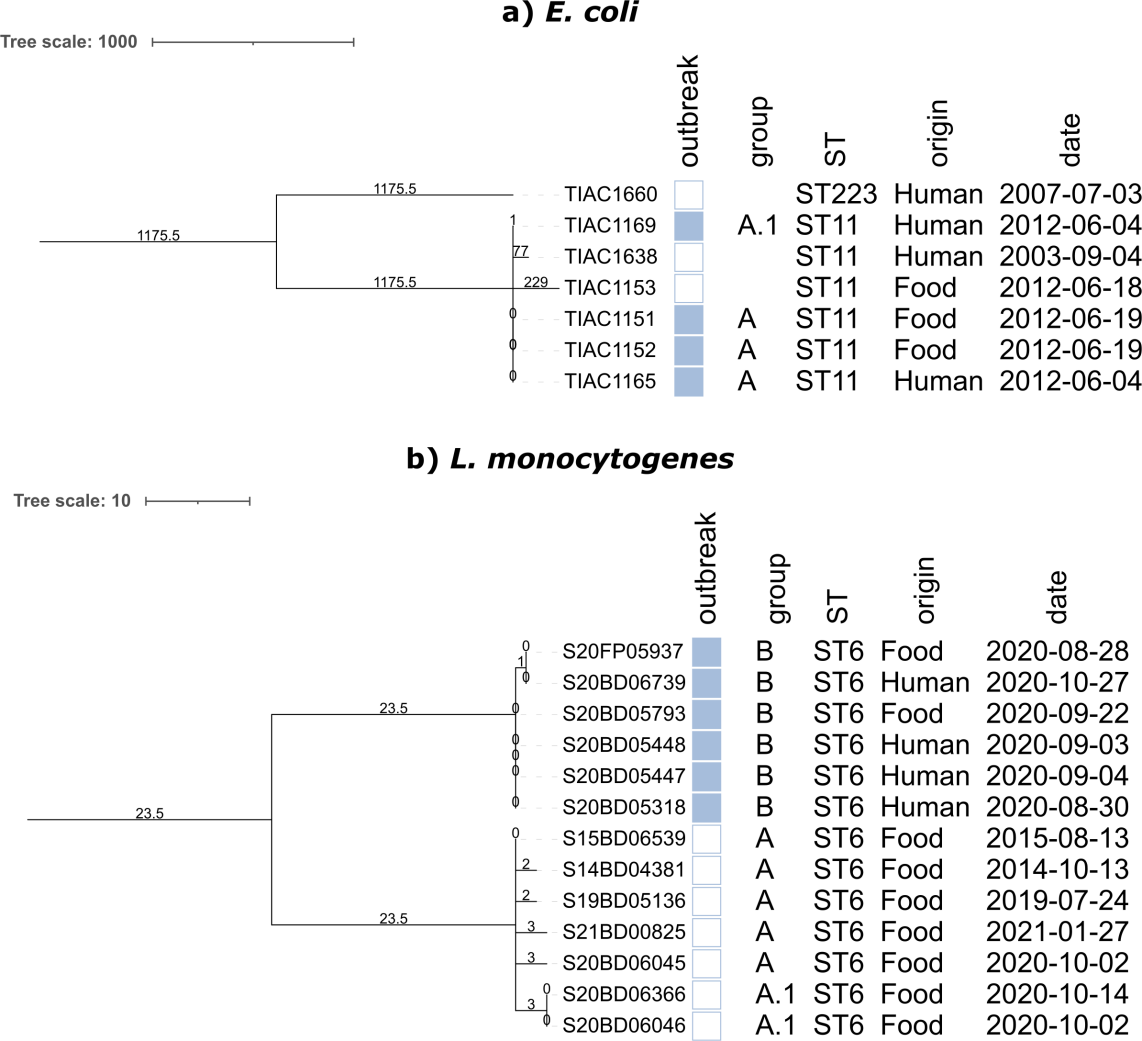
cgMLST phylogenies of the outbreak data sets: (a) *E. coli* and (b) *L. monocytogenes*. Minimum spanning tree for the *E. coli* (panel a) and *L. monocytogenes* (panel b) isolates constructed from the cgMLST results of the hybrid assemblies. Branch lengths and the scale bar are expressed as number of allelic differences. The same phylogenies are visualized as networks in Fig. S27 and S28 for *E. coli* and *L. monocytogenes*, respectively. Annotations are (from left to right) outbreak status (a filled rectangle indicates that the isolate was part of the outbreak), phylogenetic group (used to evaluate SNP distances), sequence type, sample origin, and collection date. For *E. coli*, phylogenetic group A corresponds to the three outbreak isolates that were identical. The A.1 isolate is also part of the outbreak but differs from the other phylogenetic group A isolates by a single allele. For *L. monocytogenes*, phylogenetic groups A and A.1 consist out of the seven isolates, which are not linked to the six outbreak isolates (phylogenetic group B). The isolates in phylogenetic group A are genomically very similar but not identical. Phylogenetic group A.1 consists of two isolates that were identical and differ between two and six alleles from the other isolates of phylogenetic group A. Note that the outgroup isolate S18BD0118 has been omitted from the *L. monocytogenes* phylogeny to highlight the diversity within phylogenetic group A. Abbreviations: ST, sequence type; cgMLST, core genome multi-locus sequence typing.

Samples were collected from two Belgian foodborne outbreaks caused by either *Listeria monocytogenes* or STEC. An overview of the samples is provided in Table 1, and cgMLST-based phylogenies (see Construction of cgMLST-Based Reference Topologies) are shown in Fig. 1. For STEC, six ST11 (O157:H7) isolates and one ST223 (O113:H21) isolate were selected. Four of the ST11 isolates were linked to a 2012 foodborne outbreak traced back to beef (24). Two outbreak isolates were of human origin, and the other two were of food origin. The two unrelated ST11 isolates and one ST223 isolate were included in the analysis as background to the outbreak. For *L. monocytogenes*, 14 ST6 isolates were selected, six of which were associated with a suspected foodborne outbreak that occurred in 2020. Routine surveillance using Illumina sequencing revealed very high genomic similarity between four isolates collected from patients and two isolates collected from goat cheese sampled during the same period. These six isolates are referred to as the outbreak strains in this study, with the remaining eight isolates serving as background. An additional unrelated ST155 isolate was sequenced as an outgroup. All human strains were isolated by clinical laboratories and sent to the Belgian National Reference Centers for confirmation and further typing as part of routine surveillance. Food isolates obtained during foodborne outbreak investigations and official food surveillance programs conducted by the Federal Agency for the Safety of the Food Chain were further typed by the National Reference Laboratories.

**TABLE 1:** Overview of employed isolates^*a*^

Isolate	Species	ST^*b*^	Origin	Outbreak
S14BD04381	*L. monocytogenes*	ST6	Food	No
S15BD06539	*L. monocytogenes*	ST6	Food	No
S17BD00188	*L. monocytogenes*	ST155	Human	No
S19BD05136	*L. monocytogenes*	ST6	Food	No
S20BD05318	*L. monocytogenes*	ST6	Human	Yes
S20BD05447	*L. monocytogenes*	ST6	Human	Yes
S20BD05448	*L. monocytogenes*	ST6	Human	Yes
S20BD05793	*L. monocytogenes*	ST6	Food	Yes
S20BD06045	*L. monocytogenes*	ST6	Food	No
S20BD06046	*L. monocytogenes*	ST6	Food	No
S20BD06366	*L. monocytogenes*	ST6	Food	No
S20BD06374	*L. monocytogenes*	ST6	Food	No
S20BD06739	*L. monocytogenes*	ST6	Human	Yes
S20FP05937	*L. monocytogenes*	ST6	Food	Yes
S21BD00825	*L. monocytogenes*	ST6	Food	No
TIAC1151	*E. coli*	ST11	Food	Yes
TIAC1152	*E. coli*	ST11	Food	Yes
TIAC1153	*E. coli*	ST11	Animal	No
TIAC1165	*E. coli*	ST11	Human	Yes
TIAC1169	*E. coli*	ST11	Human	Yes
TIAC1638	*E. coli*	ST11	Human	No
TIAC1660	*E. coli*	ST223	Human	No

^*a*^ Overview of the isolates used in this study. The column “Outbreak” indicates whether the isolate was confirmed to be part of the outbreak solely based on Illumina cgMLST cluster analyses.

^*b*^ ST, sequence type.

### DNA extraction

For generation of *E. coli* Illumina data sets, the Genomic-tip 20/G kit DNA extraction was used, as described elsewhere (24, 25). The DNA extraction of the *E. coli* ONT data sets was performed using the Maxwell RSC Instrument (Promega, WI, USA) according to the manufacturer’s instructions. For the Illumina and ONT sequencing of the *L. monocytogenes* isolates, genomic DNA was also extracted from pure bacterial cultures using the Maxwell RSC Instrument (Promega), except for the Illumina sequencing of the *L. monocytogenes* isolates collected from humans, for which the MagCore Genomic DNA Bacterial Kit (RBC Bioscience, New Taipei City, Taiwan) was used instead. DNA concentration and purity were assessed using the dsDNA high-sensitivity and broad-range assay kits for the Qubit 4 fluorometer (Thermo Fisher Scientific, Schwerte, Germany) and the NanoDrop 2000 spectrophotometer (Thermo Fisher Scientific), respectively. All kits were used according to the manufacturer’s instructions.

### Whole-genome sequencing

For *E. coli*, short-read Illumina data sets for the isolates from a previous study were used (25). Short-read DNA libraries for the *L. monocytogenes* samples were prepared using the Nextera XT DNA library preparation kit (Illumina, San Diego, CA, USA) according to the manufacturer’s instructions. Sequencing was performed on an Illumina MiSeq sequencer using the V3 chemistry, obtaining 250-bp paired-end reads, aiming for a theoretical coverage of 60× per isolate based on the expected genome size of ~3 Mbp for *L. monocytogenes*. The seven *E. coli* strains and the 14 *L*. *monocytogenes* isolates were each multiplexed per species on a single R9 and R10 flow cell, for a total of four flow cells. The difference in the number of multiplexed isolates per flow cell between the two species is explained by the smaller genome size of *Listeria monocytogenes* (~3 Mb) compared to STEC (~5.5 Mb). Long-read ONT R9 DNA libraries were generated using the SQK-LSK109 ligation sequencing kit in combination with the EXP-NBD104 and EXP-NBD114 native barcoding multiplexing kits (all manufactured by Oxford Nanopore Technologies, Oxford, UK). ONT R10 DNA libraries were prepared using the SQK-NBD114.24 multiplex enabled ligation sequencing kit (Oxford Nanopore Technologies). The protocol started with 1 µg of input DNA per isolate for R9 sequencing and 400 ng for R10 sequencing. The optional step of shearing the DNA into 8-kb fragments using Covaris G tubes was omitted. Sequencing was performed on a GridION instrument for 72 hours using R9.4.1 and R10.4.1 flow cells for R9 and R10 sequencing, respectively.

### Data preprocessing, quality filtering, and read mapping

#### Illumina data

Raw short reads were trimmed using Trimmomatic (v.0.38) (26) with the following options: “LEADING” set to 10, “TRAILING” set to 10, “SLIDINGWINDOW” set to “4:20,” “MINLEN” set to 40, and “ILLUMINACLIP” set to “NexteraPE-PE.fa:2:30:10.” Processed reads were mapped to the reference genome using Bowtie (v.2 2.4.1) (27) with the “--end-to-end” and “--sensitive” options enabled. Reference genomes of the same sequence type (ST) were selected. For *E. coli*, the Sakai O157:H7 genome (RefSeq accession NC_002695.2) was used. The reference genome for *L. monocytogenes* was obtained by querying the Institut Pasteur *Listeria monocytogenes* isolate database for ST6, selecting the LM09-00372 strain (RefSeq accession GCF_001565435.1).

#### Oxford Nanopore Technologies data

Dorado (v.0.3.2) (available at https://github.com/nanoporetech/dorado) was used for base calling, using the “dna_r9.4.1_e8@sup-v3.6” and “dna_r10.4.1_e8.2_400bps_sup@v4.2.0” models for the R9 and R10 data, respectively. For R9 data, the “dorado basecaller” command was used, while for R10, the “dorado duplex” command was used. For the data sets sequenced using R10 chemistry, the duplex yield was calculated by multiplying the number of duplex bases by two and dividing it by the number of simplex bases. Reads were then de-multiplexed using Guppy (v.6.4.6) (available at https://id.customers.nanoporetech.com) with the “--trim-adapters” option enabled and other options left at their default values. The resulting FASTQ files were filtered using the “seq” command of seqkit (v.2.3.1) (28), removing reads with an average quality less than 7 or shorter than 500 bp. Reads were mapped to the reference genome using Minimap2 (v.2.24) (29) with the “preset” option set to “map-ont.” The same reference genomes were used as for the Illumina data.

### Construction of cgMLST-based reference topologies

Hybrid assemblies were generated following the recommendations of Wick et al. (30) for automating the generation of long-read first hybrid assemblies. First, the R10 reads were assembled using Flye (v.2.9.1), with the “--nano-corr” and “--no-alt-contigs” options enabled (31). The resulting assemblies were polished using the “consensus” and “stitch” functions of Medaka (v.1.7.3) (available at https://github.com/nanoporetech/medaka) with the “r1041_e82_400bps_sup_g632” model and the R10 reads as input. Two polishing steps were then performed using the trimmed Illumina data. BWA (v.0.7.17) (32) was used with the “-a” option enabled to map the reads to the draft assemblies. Forward and reverse reads were mapped separately. The Polypolish (v.0.5.0) (33) ‘Insert-size-filter’ script was used with default options to filter alignments based on insert size. Polypolish (v.0.5.0) (33) was then used with the “--min-depth” option set to 5 for the first round of polishing. A second round of polishing was performed using POLCA (v.4.1.0) (34) with default options.

The hybrid assemblies were then used to construct cgMLST-based reference topologies for both STEC and *L. monocytogenes*. Loci were called using a blast-based approach described previously (35). A minimum spanning tree was constructed from the allele matrix using GrapeTree (v.2.2) (36) with the “method” parameter set to “MSTreeV2.” The cgMLST schemes were obtained from EnteroBase on 23 July 2023 and BIGSdb-Pasteur on 25 June 2023 for *E. coli* and *L. monocytogenes*, respectively (37, 38).

### Variant calling and filtering and phylogenetic tree reconstruction

#### PACU workflow

**Fig 2:**
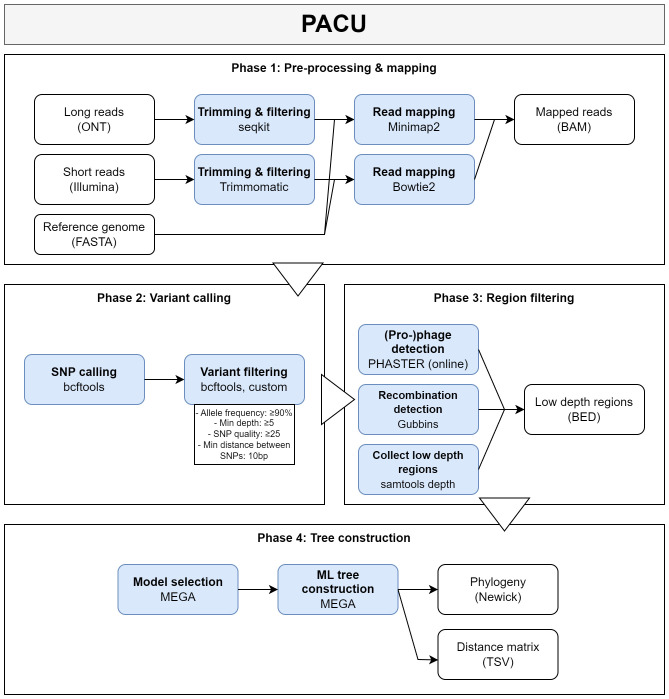
Schematic representation of the PACU SNP phylogeny workflow. This figure shows the architecture of the PACU SNP phylogeny workflow. The different analysis steps are shown in blue boxes with the primary tool(s) listed in the second row. The output and input files are shown in white boxes. Note that some steps have been simplified for clarity, as they consist of multiple command line calls and/or additional programmatic processing. The steps have been grouped according to the main phases of the analysis as indicated by the outer rectangles. The triangles indicate the order of the phases. Abbreviations: ML, maximum likelihood; ONT, Oxford Nanopore Technologies.

The BAM files resulting from read mapping were used as input for the whole-genome SNP workflow shown in Fig. 2, which is freely available on GitHub (https://github.com/BioinformaticsPlatformWIV-ISP/PACU) and as a web service in the Galaxy instance of our institute (https://galaxy.sciensano.be/, registration required). The workflow starts by checking the quality of the input BAM files by calculating the median coverage and the fraction of the reference genome that is covered using the depth function of samtools (v.1.17) (39). A warning is displayed for data sets with a median coverage of less than 20× or that cover less than 95% of the reference genome. Data sets that did not meet these quality criteria were omitted from this study. Afterward, variants were called using the “mpileup” function followed by the “call” function of bcftools (v.1.17) (39). The “--skip-variants” option was set to “indels”; the “--ploidy” option was set to “1”; and the “--consensus-caller” option was enabled. The “--prior” option was set to 0.0011 and 0.01 for Illumina and ONT data, respectively. Higher values of this option increase the sensitivity of the SNP calling, and the tool recommends using 0.01 for ONT data. Variants were filtered using the bcftools (v.1.17) “filter” command with the “--soft-filter” option enabled (i.e., variants are retained in the VCF files, but with an annotated “FILTER” column), masking variants with an allele frequency below 66%, depth below 5, or SNP quality below 50. An in-house script was then used to soft-filter SNPs that were located within 10 bases of another SNP. The unfiltered SNPs were applied to the reference genome using the “consensus” command of bcftools (v.1.17) (39). The resulting updated consensus sequences were merged into a single FASTA file, which was used as input to Gubbins (v.3.1.4) (40) to detect recombinant regions. The “sort” and “merge” functions of BEDtools (v.2.27.1) (41) were used to convert the Gubbins output file to BED format. Low-depth regions were identified using the “depth” function of samtools (v.1.17) with the minimum mapping quality set to 5 to exclude secondary alignments (39). Only the positions for which each data set had at least 5× depth were retained for the SNP analysis. The online PHASTER (42) tool was used to identify prophages in the reference genome. The “multiinter” function BEDtools (v.2.27.1) (41) was used to merge the BED files containing low-depth regions, recombinant regions, and phage regions into a single BED file. SNPs located in these regions were removed from the VCF files using the “filter” command of bcftools (v.1.17) (39). The resulting VCF files were used to construct a SNP matrix using a custom script. The soft-filtered variants were replaced by “N” in the construction of the SNP matrix. Maximum likelihood phylogenies were generated using MEGA (v.10.0.4) (43) with the “gaps/missing data treatment” option set to “complete deletion,” the “branch swap filter” set to “very weak,” the number of bootstrap replicates set to “100,” and the “ML heuristic method” set to “SPR3.” The best fitting nucleotide substitution model was determined using the “model selection” analysis of MEGA (v.10.0.4) with the same parameters. The resulting phylogenies were visualized using FigTree (available at https://github.com/rambaut/figtree). All phylogenies were midpoint rooted, unless otherwise stated.

#### Determining variant filtering thresholds

The SNP filter cut-off values were determined by a systematic comparison with the unfiltered SNPs called in the Illumina data. For each candidate filter, the values for true SNPs (i.e., detected in the Illumina data) and false SNPs (i.e., not detected in the Illumina data) were plotted. Positions that were called as multi-allelic in the Illumina data were not included in this analysis. Thresholds were then chosen to maximize the number of false SNPs removed while retaining (almost) all true SNPs. This evaluation was restricted to the SNPs in regions that passed the region filtering. Both species were considered for this analysis in order to obtain cutoffs that are generally applicable regardless of species. The following candidate filters were evaluated: depth at SNP position (“DP” tag), allele frequency (calculated from the “DP4” tag), SNP quality, and, where available, strand bias *P* value (based on the “PV4” tag).

#### Running times

The execution time of each step in the complete workflow was measured for all four ONT runs. The workflow was executed with eight threads on a virtualized server with an AMD EPYC 7452 32-core processor and 236 GB of available RAM. Base calling and de-multiplexing were performed using graphics processing unit (GPU) acceleration on a single NVIDIA GeForce RTX 3090 with 24 GB of internal memory. There were no other analyses running on the server during the benchmark. Note that this benchmark does not include the generation of the BED file with phages using PHASTER, which depends on the availability and job load of the web service, although this step only needs to be performed once per reference genome.

### Performance evaluation

The performance of the SNP phylogeny construction using the different technologies/chemistries was evaluated using several strategies. First, phylogenies were constructed including all isolates sequenced using the same technology/chemistry for both species (e.g., all ONT R9 *E. coli* isolates). These phylogenies were then compared by (i) visual comparison of the topologies, (ii) overlap between detected variants, (iii) the size of the SNP matrices, (iv) pairwise distances between predefined phylogenetic groups, and (v) calculation of the Kendall-Colijn tree similarity metric. The Kendall-Colijn metric compares two trees by calculating Euclidean distances from tip to root with a coefficient *λ* to give more weight to either topology (*λ* = 0) or branch length (*λ* = 1) (44), which was set to 1 (13). As the metric is influenced by the topology of the tree, the number of leaves, and the branch lengths, it is not suitable for comparing trees with different leaves. Lower values of the lambda parameter were not considered, because we did not want to bias the metric by the placement of the very closely related outbreak isolates.

Second, mixed phylogenies were constructed that combined the different technologies/chemistries in two different approaches. In the first approach, mixed phylogenies were constructed that contained all data sets generated by two different technologies/chemistries to assess concordance in the placement of isolates between technologies/chemistries (i.e., each isolate was represented by two separate data sets generated by a different technology/chemistry). In the second approach, mixed phylogenies that contained only a single data set per isolate were generated, whereby the technology/chemistry used could differ between different isolates included in the same analysis. This approach mimics real-world outbreak scenarios, where isolates are often sequenced by different laboratories that may use different sequencing devices and/or technologies. For the second approach, 10 replicate phylogenies were generated for all possible combinations of R9, R10, and Illumina and were compared to the Illumina-only phylogeny. The selected data sets for these replicates are listed in Tables S7 and S8 for *E. coli* and *L. monocytogenes*, respectively. Performance was evaluated using the criteria listed in the previous paragraph, with the exception of the Kendall-Colijn metric, which could not be calculated for the mixed phylogenies in the first approach because they contained twice or three times the number of samples compared to the Illumina reference topology. Note that hybrid approaches, where data sets generated using Illumina and ONT are combined to reconstruct a genome for a single isolate, were not considered for the phylogenomic analysis.

### Evaluation of required sequencing time for ONT R9 and R10

For both R9 and R10, the workflow performance was characterized in function of the sequencing time by generating sub-sampled data sets containing reads sequenced at 2, 4, 8, 12, 16, 20, 24, 28, 32, 36, and 40 hours. Read identifiers for each time point were retrieved from the sequencing summary file, and reads were extracted from the full FASTQ files using the “subseq” function of seqtk (v.1.4) (available at https://github.com/lh3/seqtk). The resulting data sets were analyzed as described in the previous sections, except that data sets with a median depth of less than 20× were retained in these phylogenomic analyses, although they would normally be flagged as low quality in the standard workflow. Performance at the different time points was assessed by comparing the resulting phylogeny with the phylogeny generated using the Illumina data, which was considered the “gold standard.” As the number of active pores varies between flow cells, the same analysis was repeated based on the total number of reads sequenced. Sub-sampled data sets of 100,000–1,000,000 reads were generated at intervals of 100,000 reads. The performance evaluation was performed in the same manner.

## RESULTS

### WGS data quality and yield

Read trimming and filtering statistics for the Illumina data are shown in Table S2. The number of read pairs ranged from 279,281 to 707,090, with a median of 484,951. The median number of read pairs after trimming and filtering was 442,898. Statistics for the four ONT runs were obtained from the unfiltered reads and are provided in Table S3. For both species, the median read lengths were longer for the R9 runs compared to the R10 runs. For *E. coli*, the median read lengths were 1,027 and 811 bp for the R9 run and R10 runs, respectively. For *L. monocytogenes*, the median read lengths were 1,717 and 932 bp for the R9 and R10 runs, respectively. The median read quality was higher for the R10 data, with 16.1 for *E. coli* and 17.0 for *L. monocytogenes*, compared to 12.8 for *E. coli* and 13.4 for *L. monocytogenes* for the R9 data. For the *E. coli* runs, the number of reads generated was slightly lower for the R9 run compared to the R10 run (i.e., 4,465,213 and 5,099,405 reads), but the longer reads resulted in a higher yield for the former, with 1.09 × 10^10^ and 7.95 × 10^9^ total bases for the R9 and R10 runs, respectively. For the *L. monocytogenes* runs, the R10 run had a higher number of reads and yield, corresponding to 3,876,476 reads and 6.65 × 10^9^ total bases, compared to 1,809,723 reads and 5.96 × 10^9^ bases for the R9 run. The duplex yield for the R10 runs was only 5.24% for *E. coli* and 10.46% for *L. monocytogenes*. The duplex yield was not sufficient to cover the genome to an adequate depth, and the duplex-only data sets were not further considered for the subsequent SNP analysis.

### Read mapping

Post-filtering read mapping statistics are provided in Table S4 and shown in Fig. S1. For *E. coli*, the median depths per isolate were 38.5×, 166×, and 141.5× for Illumina, R9, and R10, respectively. For *L. monocytogenes*, the median depths were 58×, 78×, and 103.5× for Illumina, R9, and R10, respectively. The variation in depth was higher for the ONT runs, especially for *L. monocytogenes*. For two isolates, S15BD06539 (9×) and S20BD05448 (18×), the median depth in the R9 run was below 20×, which was considered the minimum for subsequent SNP analysis. These isolates were therefore omitted from the SNP analysis, including the data sets generated by Illumina and ONT R10 that were above the 20× threshold. For *E. coli*, the median mapping rates were 100%, 97.6%, and 97.4% for Illumina, R9, and R10, respectively. For *L. monocytogenes*, the mapping rate for ONT was slightly lower, with median values of 100%, 93.9%, and 93.6% for Illumina, R9, and R10, respectively. For all data sets, the breadth of coverage of the reference genome was above 95%. For the outgroups with a different ST (i.e., TIAC1660 for *E. coli* and S17BD00188 for *L. monocytogenes*), the fraction was lower due to the greater genomic distance to the reference genome. Note that the other metrics may also be influenced by the relatedness to the reference genome.

### Variant calling and filtering

#### Determining variant filtering thresholds

Plots of the variant filtering metrics for true SNPs and false SNPs, compared to the Illumina data, are shown in Fig. S2 through S5. For both ONT R9 and R10, the depth values of true and false SNPs were mostly overlapping and therefore not suitable for distinguishing true SNPs from false SNPs, although a minimum depth value of 5 was enforced to mask SNP calls based on a very small number of bases. For the R9 and R10 data sets, the allele frequency of the true SNPs was on average higher than that of the false SNPs. The variation was greater for the R9 data sets, resulting in a greater overlap between true and false SNPs. A threshold value of 66% was chosen to retain the majority of true SNPs while removing the majority of false SNPs, particularly in the R9 data sets. Notably, some of the true SNPs in the *E. coli* Illumina data sets had very low allele frequencies (i.e., below 50%), which is a limitation of using the unfiltered Illumina SNPs as a reference standard. For all technologies/chemistries, the SNP quality of the true SNPs peaked at the maximum value (i.e., 255). The variation was greater for the values observed in the R9 data sets, resulting in overlap between the true and false SNPs. A threshold value of 50 was selected, which removed the vast majority of false SNPs while limiting the number of true SNPs removed. Finally, the *P* value of the strand bias was evaluated. Interestingly, the vast majority of the false SNPs detected in the R9 data had a (near) 0 *P* value, indicating that the alternative bases were mostly restricted to one of the two strands. Although the observed values for the false SNPs were generally lower than those for the true SNPs, it was not possible to find a threshold value that did not exclude a substantial proportion of the true SNPs. Therefore, this metric was not used for variant filtering.

#### Variant calling and filtering statistics

The numbers of SNPs before and after filtering are listed in Table S5 and visualized in Fig. S8. For *E. coli*, the median numbers of SNPs before filtering were 215, 218, and 213, for Illumina, R9, and R10, respectively. Note that these numbers were calculated after removing problematic regions as described in Variant Calling and Filtering and Phylogenetic Tree Reconstruction. After filtering, medians of 205, 210, and 210 SNPs remained for Illumina, R9, and R10, respectively. Similar results were observed for *L. monocytogenes*, with median of 97, 108, and 96 SNPs before, and medians of 94, 94, and 95 SNPs after filtering for Illumina, R9, and R10, respectively. These trends observed in the median number of SNPs across all isolates observed for a species for a given sequencing technology/chemistry were also consistent at the level of the individual isolates. In summary, the number of SNPs before and after filtering was very similar for the three technologies/chemistries.

#### Overlap between detected SNPs

**Fig 3:**
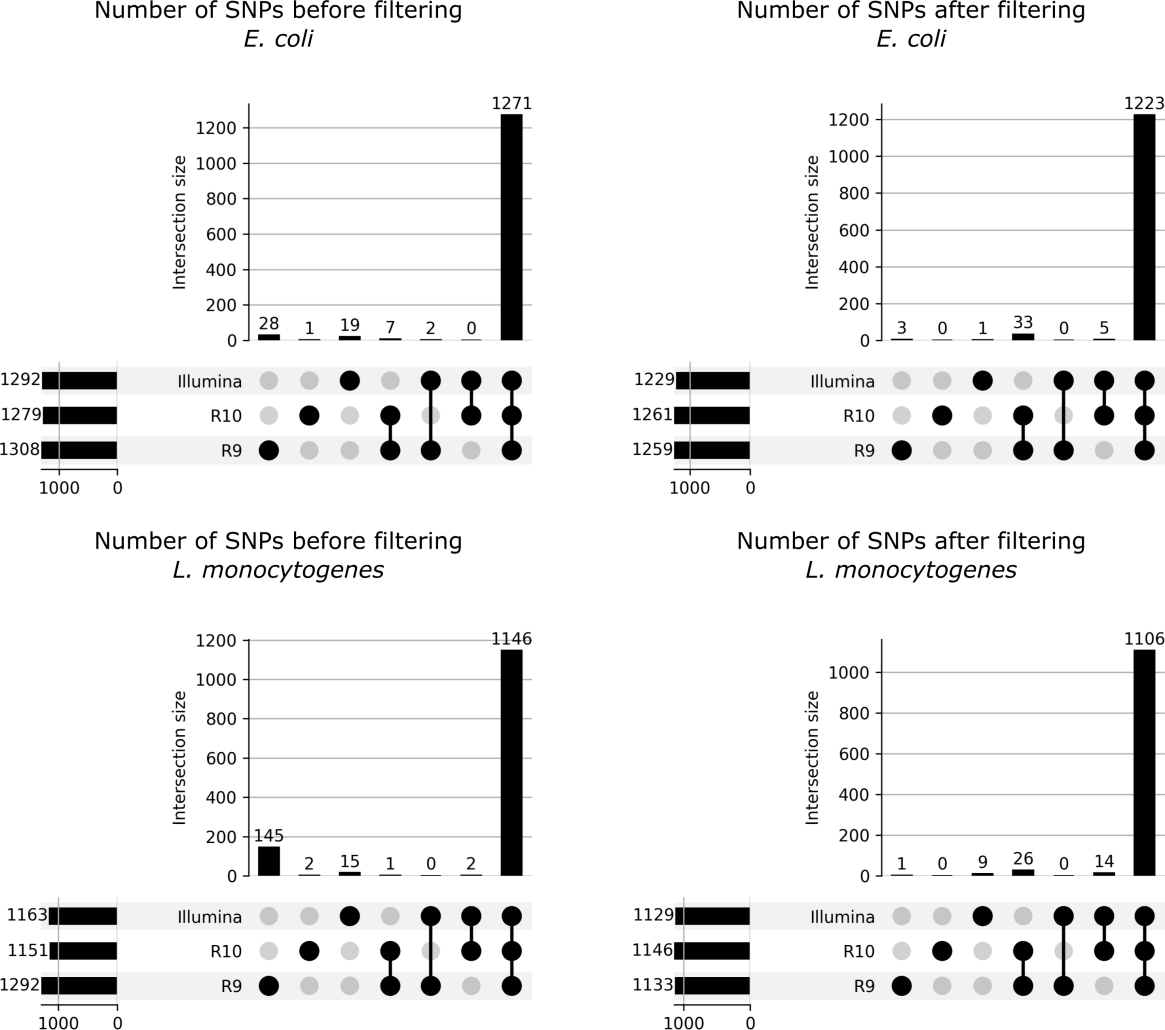
UpSetPlots showing the overlap in SNPs between different sequencing technologies/chemistries before and after filtering. Plots show the size of the intersection of the detected SNPs between the sequencing technologies/chemistries across all isolates. The results for *E. coli* and *L. monocytogenes* are shown in the top and bottom rows, respectively. The results before and after SNP filtering are shown on the left and right sides, respectively. The size of the vertical bars indicates the size of the intersection between technologies/chemistries, as indicated by the black dots and connecting lines below the bars. For example, the leftmost bar in each sub-plot indicates the number of SNPs uniquely detected in the R9 data sets across all isolates of the corresponding species, whereas the rightmost bar in each sub-plot indicates the number of SNPs shared for both the Illumina, R9 and R10 data sets across all isolates of the corresponding species. The horizontal bars represent the total number of SNPs detected for each technology/chemistry. SNPs were considered to overlap between technologies if the same alternate base at the same chromosomal position was called in the corresponding data sets. Combinations with no overlap (i.e., zero SNPs uniquely shared between the corresponding sequencing technologies/chemistries) were still shown to ensure visual consistency between sub-plots. Note that these values were calculated based on the SNPs called in regions that passed region filtering, as described in Variant Calling and Filtering, and Phylogenetic Tree Reconstruction. Plots were generated using the UpSetPlot (v.0.8.0) python package.

The overlap between the detected SNPs (i.e., finding the same alternate allele at the same chromosomal position) for the data sets generated by the different sequencing technologies/chemistries before and after filtering is shown in Fig. 3. Before filtering, although the majority of all SNPs was shared between the three sequencing technologies/chemistries for both *E. coli* and *L. monocytogenes*, the largest number of unique variants was always exhibited by R9, whereas the number of SNPs uniquely detected in either the R10 or Illumina data sets was generally lower. After filtering, the vast majority of SNPs were still shared between the three sequencing technologies/chemistries for both *E. coli* and *L. monocytogenes*. For *E. coli*, the number of SNPs detected by both R10 and R9 (*n* = 33) was higher than those detected by both R10 and Illumina (*n* = 5). Similarly, the number of SNPs shared between R10 and Illumina for *L. monocytogenes* (*n* = 14) was lower than the number of SNPs shared between R9 and R10 (*n* = 26). For both species, the number of SNPs shared between Illumina and R9 that were not detected in the R10 data sets was zero. These results indicate that the SNPs detected in the R10-R9 and R10-Illumina data sets tended to overlap more than those between the R9 and Illumina data sets. However, it should be noted that the SNPs were restricted to genomic regions that were sufficiently covered by three technologies, and the number of SNPs may differ slightly when a single technology is considered in isolation.

### Phylogenomic investigation

#### Single-technology/chemistry phylogenies

**Fig 4:**
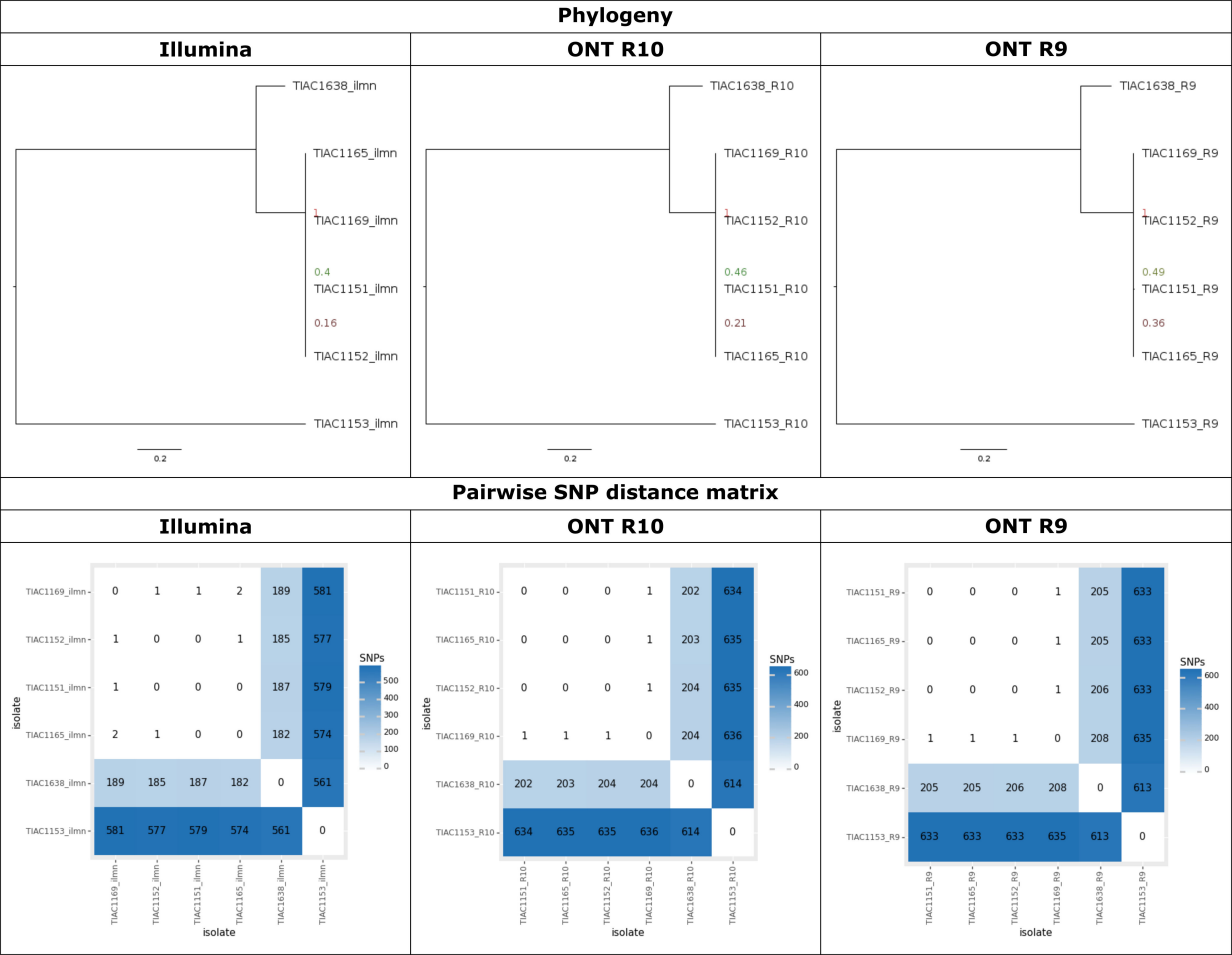
Comparison between *E. coli* phylogenies generated with Illumina, R10, and R9 data. The results of the phylogenetic analysis for Illumina (left panel), R10 (middle panel), and R9 (right panel) data sets. The top row shows the phylogenetic trees. Branch lengths and the scale bar are expressed as average number of substitutions per site. Node labels indicate the bootstrap support for the corresponding node. The bottom row shows the pairwise SNP distances.

**Fig 5:**
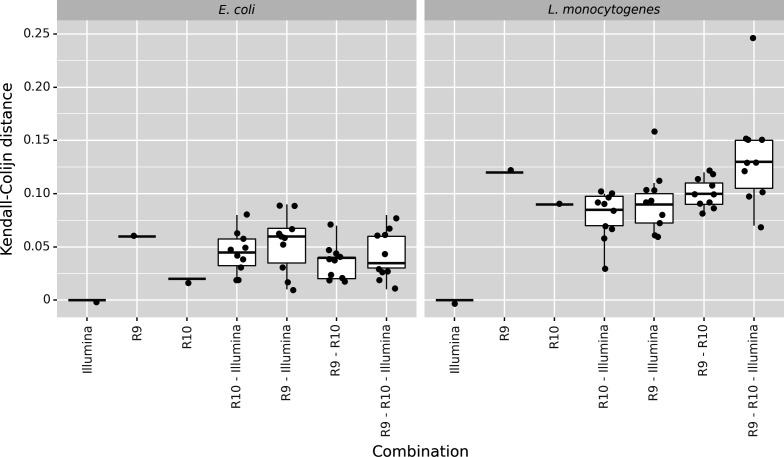
Similarity of single- and mixed-technology/chemistry phylogenies compared to the Illumina reference phylogeny. The *x*-axis shows the technologies or chemistries that were included in the phylogeny. The *y*-axis shows the Kendall-Colijn distance to the Illumina reference phylogeny. Each point represents a single comparison. The mixed-technology/chemistry combinations represent phylogenies whereby for different isolates, data sets generated by different technologies were selected from the pool of available data sets according to the label on the *x*-axis (10 phylogenies were generated each time by randomly selecting one of the corresponding technologies for each isolate).

Phylogenies including all data sets generated by the same technology/chemistry separately were first constructed for both species. Side-by-side comparisons for the different technologies/chemistries are shown in Fig. 4 for *E. coli* and in Fig. S9 for *L. monocytogenes*. In general, the tree topologies were nearly identical between the three technologies/chemistries for both species, with the outbreak isolates always clustering separately from the unrelated isolates on branches with high bootstrap support. The topologies and SNP distance matrices of the *E. coli* phylogenies for the three technologies/chemistries were almost identical (Fig. 4). However, there were minor differences in the SNP distances to the unrelated isolates, which tended to be slightly larger in the R9 and R10 data sets (Fig. S12). Minor differences were also observed in the pairwise distances between outbreak isolates, with at most a single SNP difference between the Illumina and ONT R9/R10 data. The SNP matrix contained 779 and 778 positions for R9 and R10, respectively, compared to 711 positions for Illumina. The proportion of Ns (i.e., filtered SNPs) in the SNP matrices was relatively low (<0.5%) for all three technologies/chemistries. The larger SNP matrices for ONT R9/R10 may have been a consequence of the longer reads, which allowed covering genomic regions that were not covered by the short-read Illumina data (Fig. S10). The Kendall-Colijn distance to the Illumina reference phylogeny indicated that the resulting R10 phylogeny was more similar to the Illumina reference phylogeny than the R9 phylogeny (Fig. 5). Nevertheless, as shown in the phylogenies and the pairwise SNP distances in Fig. 4, the three phylogenies were almost identical. For *L. monocytogenes*, the phylogenies for the three technologies/chemistries and the pairwise SNP distance matrices were also almost identical (Fig. S9). In contrast to the *E. coli* phylogenies, the SNP distances between outbreak isolates (i.e., phylogenetic group B) and between isolates of different phylogenetic groups were slightly lower for the R9 data sets (Fig. S13). The proportions of the reference genome that were omitted from the SNP analysis were much lower than those for *E. coli* (Fig. S10). Consequently, the differences between the technologies/chemistries were also much smaller, resulting in nearly identical numbers of positions considered for the SNP analysis. However, due to fewer SNPs passing filtering in the R9 data sets, the size of the R9 SNP matrix (165 positions) was smaller than that of R10 and Illumina, where the SNP matrices contained 175 and 173 positions, respectively (Fig. S11). Similar to the *E. coli* phylogenies, the Kendall-Colijn distance to the Illumina reference phylogeny indicated that the resulting R10 phylogeny was more similar to the Illumina reference phylogeny than the R9 phylogeny (Fig. 5). In summary, these results suggest that the ONT data sets allowed SNP calling over a larger proportion of the reference genome compared to the Illumina data sets, especially for *E. coli*. They also indicate that, on average, more SNPs called in the R9 data sets are filtered out. The R10 data allow covering a similar proportion of the genome as the R9 data, but more of the SNPs are retained after filtering, resulting in slightly larger (i.e., for *E. coli*) or more or less the same size (i.e., for *L. monocytogenes*) SNP matrices as Illumina. In addition, comparison of the resulting phylogenies using the Kendall-Colijn metric indicates that the phylogenies generated using R10 data are more similar to the Illumina reference phylogeny than those generated using R9 data.

#### Mixed-technology/chemistry phylogenies

**Fig 6:**
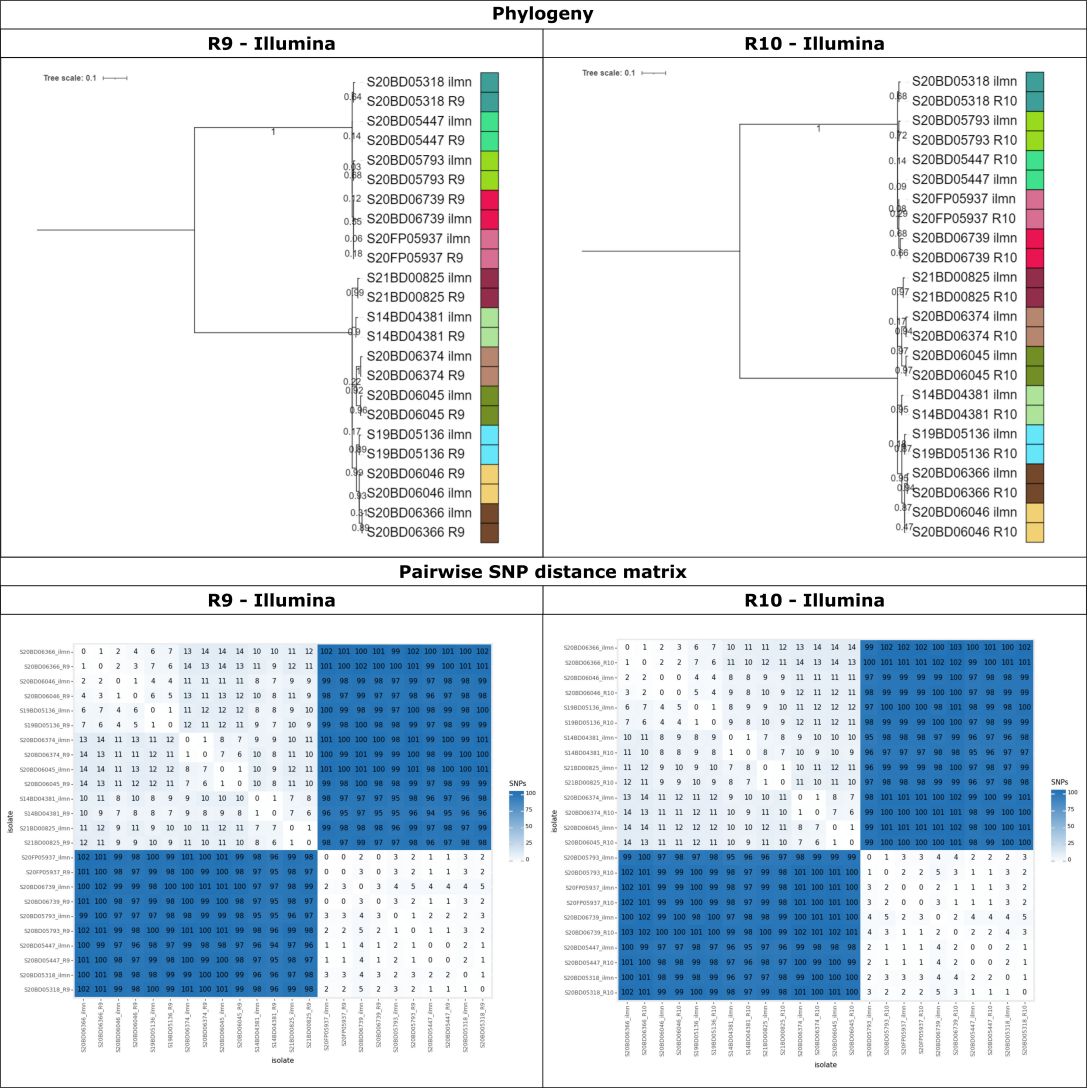
Mixed SNP phylogeny of the *L. monocytogenes* Illumina and R9/R10 data sets. These plots show the maximum likelihood SNP phylogeny including all *L. monocytogenes* data sets sequenced with Illumina and R9 (left panel), and Illumina and R10 (right panel). The top row shows the maximum-likelihood phylogenies. Branch lengths and the scale bar are expressed as average substitutions per site. Node labels indicate the bootstrap support for the corresponding node. Replicates of the same isolate sequenced with different technologies are indicated by the colors on the right side of the phylogeny (the “R9,” “R10,” and “ilmn” suffixes, respectively, refer to ONT R9, ONT R10, and Illumina). The bottom row shows the pairwise SNP distance matrices.

Mixed phylogenies containing data sets generated by two technologies/chemistries, were evaluated afterward. First, mixed phylogenies were created containing all data sets generated by two different technologies/chemistries to evaluate concordance in the placement of isolates (i.e., every isolate was represented by two separate data sets). Figure 6 shows the phylogenies for *L. monocytogenes* combining the Illumina with R9 and R10 data sets, and other combinations are shown in Fig. S14 through S21. The same general trends as for the single-technology/chemistry phylogenies were observed, with very similar phylogenetic results for the three technologies/chemistries. The same isolate always clustered within three and two SNPs for the R9 and R10 data sets compared to Illumina, respectively. Despite these small genomic differences, different data sets for the same isolate always clustered together in the mixed phylogenies. The pairwise SNP distances were very similar for the mixed phylogenies. For both species, the sizes of the SNP matrices were almost identical for all mixed phylogenies, except for the *E. coli* R9-R10 phylogeny, which was slightly larger, likely due to the larger proportion of the reference genome used for SNP calling (Fig. S10). Note that these mixed phylogenies could not be compared to the Illumina reference phylogeny using the Kendall-Colijn metric as presented in Fig. 5 because they contain a different number of isolates.

Second, mixed phylogenies were created containing only a single data set per isolate (i.e., every isolate was represented by a data set of a single technology/chemistry). The similarity of these mixed phylogenies compared to the Illumina reference phylogenies is illustrated in Fig. 5. For *E. coli*, the R9-Illumina and R10-Illumina mixed phylogenies were very similar to the Illumina reference phylogeny, with median Kendall-Colijn distances of 0.054 and 0.045, respectively. The phylogenies were also stable, as indicated by the relatively small standard deviations of 0.027 and 0.019, respectively (i.e., the replicate phylogenies have comparable similarity to the reference phylogeny, regardless of which isolate is sequenced by Illumina/ONT). The R9-R10 mixed phylogenies were generally the most similar to the reference phylogeny, with a median Kendall-Colijn distance of 0.036. For *L. monocytogenes*, the Kendall-Colijn distances of the R9-R10, R9-Illumina, and R10-Illumina phylogenies were in the same range, albeit a bit higher than for *E. coli*. For the mixed *L. monocytogenes* phylogenies, some outliers were observed where the distance of the resulting topology to the reference Illumina phylogeny was much higher than for other replicates of the same combinations. On average, the distances of the phylogenies combining two technologies/chemistries were comparable to the distance of the R10 phylogeny, while the distances of the phylogenies combining all three technologies/chemistries were comparable to that of the R9 phylogeny.

#### Running times

The execution time of the complete workflow to generate single-technology phylogenies for ONT R9/R10, including base calling and other preprocessing steps, is shown in Fig. S22 for all runs. The complete workflow took an average of 8 hours and 26 minutes, with the base calling accounting for the majority of this time. The total time was also highly dependent on the yield of the sequencing run. Without the base calling, the workflow took an average of 2 hours and 1 minute. The SNP phylogeny workflow accounted for only a very small fraction of the total time, with an average of 5 minutes per run.

### Performance evaluation in function of sequencing time

We evaluated the required sequencing time on three different levels. First, the number of reads sequenced in function of the sequencing time for the four ONT runs is shown in Fig. S23. For both species, the R10 run reached a higher throughput earlier, with half of the reads already sequenced after ~10 hours, while this took ~15 to 18 hours for R9. Second, however, as the total sequencing yield is a function of both the number of reads sequenced and their individual length, we determined the minimum sequencing time to achieve sufficient coverage for SNP-based phylogenomic analysis using 20× as a threshold. The results are shown in Fig. S24. As explained in Read Mapping, only 13 of the 15 isolates were above the threshold in the full *L. monocytogenes* R9 run. For the two R10 runs, all data sets were above the threshold after only 8 hours of sequencing. For R9, sequencing had to be at least twice as long, even after excluding the two *L. monocytogenes* isolates that were below the threshold in the full run. Third, since the coverage threshold of 20× describes the minimum coverage required to run the SNP workflow but does not inform on the accuracy of the obtained final phylogenetic trees, we also evaluated the Kendall-Colijn metric. The similarity between the phylogenies constructed at different time points and the reference phylogeny is shown in Fig. S25. For *E. coli*, the phylogeny stabilized after ~12 hours of sequencing for the R10 data sets and after ~20 hours of sequencing for the R9 data sets. The variation was greater in the *L. monocytogenes* phylogenies. The R10 phylogenies stabilized after ~12 hours of sequencing. For the R9 phylogenies, the distance initially increased as the number of covered positions increased but decreased after ~16 hours of sequencing and stabilized after ~26 hours of sequencing. The Kendall-Colijn metric indicated that accurate phylogenetic results could be obtained after about half a day of R10 sequencing, whereas it took twice as long for R9. When the analysis was performed in function of the number of reads sequenced, similar results were observed (Fig. S26). However, the advantage of R10 was less pronounced because the R10 reads were shorter on average and would be sequenced faster in practice than the longer R9 reads. It should be noted that since the above analyses always only considered two runs per ONT chemistry, the conclusions may not be representative of the chemistries in general as variation in sequencing speed and the number of active pores can affect these results.

## DISCUSSION

In this study, we present a workflow for SNP-based phylogenomic investigation of bacterial outbreak isolates with adapted SNP filters for ONT data. The workflow is available as an open-source workflow on GitHub (https://github.com/BioinformaticsPlatformWIV-ISP/PACU) and can be executed from our institute’s web-based Galaxy instance (https://galaxy.sciensano.be, registration required). This workflow was evaluated on two historical outbreaks of STEC and *L. monocytogenes* to evaluate the performance of ONT R9 and R10 for this type of analysis. The R9 reads were, on average, longer than the R10 reads, allowing a larger fraction of the reference genome to be covered. The read quality was higher for the R10 data, with a difference in Phred score of ~4. The throughput per run was comparable for the R9 and R10 chemistries but varied considerably from run to run. Although coverage can have a large effect on the accuracy of SNP calling, we chose to omit normalization to simplify the analysis and to mimic a real-world scenario where isolates are typically sequenced at different depths. Moreover, the relatively high sequencing depths for ONT for the number of samples typically multiplexed by most laboratories in a single run could be considered an advantage of using ONT over Illumina, as the latter typically requires more samples to be multiplexed to achieve the same level of cost-effectiveness per run. However, the duplex yield was relatively low for the two R10 runs. Omitting the duplex reads from the FASTQ files had no notable effect on any of the R10 phylogenies, and the duplex throughput was not sufficient to cover the entire genome at an adequate depth (results not shown). Therefore, the added value of duplex base calling for this type of analysis is currently very limited. However, as chemistry and algorithms improve the duplex yield, the increased read quality may improve the accuracy of SNP calling.

The accuracy of variant callers can have a substantial impact on phylogenetic tree inference. Particularly in outbreak investigations, false-positive SNPs can have a large impact due to the limited number of true SNPs among isolates (16). Therefore, variant filtering is typically used to remove or mask low-confidence SNPs. There are numerous metrics used to filter SNPs, and workflows generally employ different strategies, but depth, allele frequency, and mapping quality are commonly used (14, 16). While threshold values have been proposed for Illumina and ONT (13, 19, 45), a thorough evaluation is still required to take into account the specifics of the variant caller, species, reference genome, etc. In this study, variant filtering thresholds were based on comparison with Illumina as the current gold standard, as has been done elsewhere (19). Before filtering, the number of SNPs uniquely detected in the R9 data sets was substantially higher than those in the Illumina and R10 data sets, indicating that variant filtering may be necessary to remove false SNPs. In general, the values for the true and false SNPs overlapped more for the R9 data sets, making it more difficult to find threshold values that removed false SNPs while retaining true SNPs. Values were chosen that resulted in an optimal split between true and false SNPs in both species for all three technologies/chemistries. Lohde et al. have recently shown that DNA modifications can result in considerable base errors when using ONT sequencing (7). The allele frequency filtering in PACU handles these cases by masking positions where the consensus base is not clearly defined, thereby limiting the impact of ambiguous positions resulting from DNA modification on cluster definitions. The *P* value for strand bias was also evaluated for variant filtering, which indicated that the many false SNPs detected in the R9 data sets were mostly confined to a single strand, as also observed elsewhere (46), which was not the case for the R10 data sets. However, many true SNPs also had low *P* values, and the metric could therefore not be used for filtering. After filtering, the number of SNPs was almost identical for the three technologies/chemistries, with the vast majority of SNPs shared between the three technologies/chemistries (Fig. 3), indicating that the variant filters removed most false SNPs. Interestingly, there was some overlap of filtered SNPs uniquely shared between Illumina-R10 and R9-R10, but there were no SNPs that passed filtering detected by the Illumina and R9 data sets that were not detected in the R10 data sets. The total number of unique SNPs (i.e., only detected by a single technology/chemistry) that passed filtering was very low (an example is shown in Fig. S6). These positions were investigated further, but no discernible common cause could be identified.

The SNP-based workflow was able to accurately reconstruct the phylogenetic topology for both species using data generated by each of the three sequencing technologies/chemistries. Outbreak isolates were always clearly separated from the unrelated isolates with high bootstrap support and long branch lengths. Small differences were observed in the size of the SNP matrices and the pairwise SNP distances between the phylogenetic groups (Fig. S11 through S13). This was due to the region filtering, which removed more of the reference genome because it could not be covered by the short-read Illumina data. For *L. monocytogenes*, the difference in the fraction of the reference genome that could be covered by short and long reads was negligible (Fig. S10), which may explain the relatively lower number of filtered SNPs detected only in the ONT data (Fig. 3). For both species, the Kendall-Colijn metric indicated that the R10 phylogeny was more similar to the Illumina phylogeny than the R9 phylogeny. Phylogenies were then constructed by integrating the data sets generated using different technologies/chemistries, which were generally very accurate. Although ideally the sequencing method for an outbreak investigation by different laboratories should be harmonized, this is often not feasible in practice. Therefore, our study evaluated the performance of combining different sequencing technologies/chemistries within the same phylogenomic analysis. When utilizing multiple data sets per isolate generated with different sequencing technologies/chemistries, for example, in the *L. monocytogenes* phylogeny combining the R10 and Illumina data sets (Fig. 6), all data sets of the same isolate generated by different sequencing technologies clustered identically (with maximum two SNP differences) with each other. For the R9-Illumina combined phylogeny, the distances between different data sets for the same isolate tended to be slightly greater and sometimes slightly impacted the clustering of isolates. Nevertheless, for both species, all mixed phylogenies were accurate and very similar to the reference phylogeny, with always high bootstrap support for the outbreak clades delineated clearly from the unrelated isolates. Similar performance was obtained for the mixed phylogenies where only a single data set was included for each isolate (Fig. 5). Within the SNP analysis using PACU of these two outbreak data sets, the R9, R10, and Illumina data sets could hence be used interchangeably, with little effect on the resulting phylogenies and SNP distances. Notwithstanding, the R10 chemistry did exhibit a small advantage over the R9 chemistry due to a higher proportion of SNPs passing filtering and fewer initial false SNP calls. While generally high concordance was observed for Illumina and ONT R9/R10, discrepant SNPs were still observed (e.g., Fig. 3). A hybrid approach combining short and long reads for SNP calls could provide a higher resolution through a consensus approach. However, this will rarely be economically viable for routine surveillance. Therefore, in our study, hybrid approaches were only considered for the assembly-based construction of the cgMLST reference topologies.

ONT sequencing offers flexibility through small sequencing devices and rapid turnaround times (47), which could be advantageous for outbreak investigations. Our results show that the phylogenies generated using R10 sequencing data stabilize after ~12 hours of sequencing. The complete bioinformatics processing took less than 12 hours for all runs (Fig. S22). Consequently, a response can typically be provided within a day of starting the sequencing, which represents a substantial reduction in throughput time compared to a full 2 × 300 bp Illumina run, although Illumina sequencing time can also be reduced substantially through various optimizations such as reduced read lengths (48) or the implementation of specialized adaptations that allow real-time base calling (48, 49). Live-base calling (i.e., base calling during sequencing) could further reduce the throughput time of ONT sequencing by performing the most time-consuming step of the workflow (i.e., base calling) at the same time as the sequencing (22). A reduction in overall processing time could also be achieved by using the rapid barcoding kit, which can produce results faster than the ligation kit used in this study. More computing resources could also reduce the overall time, as most steps can be efficiently parallelized across central processing units (CPUs) or GPUs. However, laboratories wishing to integrate R9/R10 sequencing for this type of analysis may opt to re-evaluate performance on their specific cases, as the results presented here were obtained using a limited number of flow cells without using replicate sequencing runs and a specific DNA extraction protocol. Reported values for sequencing quality and yield may therefore be subject to variation, as several other factors such as targeted species, input DNA quality, base quality scores, active pores, sequencing speed, and required sequencing depth for the number of samples multiplexed, could potentially influence these results. Consequently, thresholds for delineating clusters of related isolates should be defined on a case-by-case basis, as also discussed elsewhere (50, 51).

Other studies have previously shown that ONT sequencing is promising for the rapid identification of clusters in outbreaks. Hallgren et al. showed that the MINTyper software was able to rapidly cluster isolates with known epidemiological links using R9 sequencing (20). Their results are not directly comparable with our study because they optimized the turnaround time by using a fast base calling model. However, our results confirmed that a limited sequencing time was sufficient to cluster the outbreak isolates with high bootstrap support for the R9 data sets. In another study, Phillips et al. showed that 30 minutes of R9 sequencing (corresponding to ~10× depth) was sufficient to identify closely related *Neisseria gonorrhoeae* strains (6), but they observed issues in directly comparing Illumina and ONT results due to the lower quality of the SNPs in the ONT data. Our study highlights that direct comparison by including isolates sequenced by either Illumina or ONT in the same phylogenetic tree reconstruction is possible when using optimized variant filters. Finally, Ferreira et al. showed that ~30 hours of R9 sequencing is sufficient to confirm methicillin-resistant *Staphylococcus aureus* outbreaks (4), which is consistent with our findings that R9 phylogenies stabilize after ~26 hours of sequencing. In addition, our study demonstrates, to the best of our knowledge, for the first time that the new R10 chemistry provides an improvement for this type of analysis compared to the old R9 chemistry and leads to SNP-based outbreak results on par with Illumina sequencing.

Public health and other laboratories seeking to integrate ONT sequencing into their outbreak investigation activities can use our bioinformatics workflow, PACU, which produced highly accurate phylogenomic results on the tested data sets. In light of the still constantly changing protocols for nanopore sequencing and the variation that exists in DNA extraction methods, library preparation methods, etc., careful re-assessment of our results, however, is advised by labs wishing to implement nanopore sequencing into their activities to ensure comparable results to those presented on our two cases. With R9 sequencing soon to be phased out, future ONT sequencing will be based on the R10 or newer chemistries, but the performance evaluation of the R9 data presented here still provides valuable information for analyzing historical data generated by R9 sequencing. The proposed strategy for evaluating the performance of phylogenomic analysis can be easily applied to other data sets and workflows.

In conclusion, this study shows that R10 sequencing offers an advantage over R9 sequencing for bacterial outbreak investigation, enabling a more accurate and rapid response at a level that is comparable to Illumina sequencing. Additionally, we demonstrate that R10 sequencing and Illumina sequencing can be used interchangeably within this SNP-based phylogenomics workflow, facilitating efficient collaboration between laboratories employing different sequencing technologies when performing collaborative outbreak investigation.

## Data Availability

The data sets supporting the conclusions of this study have been deposited in the National Center for Biotechnology Information Sequence Read Archive under accession number PRJNA1014400. Individual accession numbers are provided in Table S1.

## SUPPLEMENTAL MATERIAL

Supplemental figures and tables jcm.01576-23-s0001.docx Fig. S1 to S28 and Tables S1 to S7.

ASM does not own the copyrights to Supplemental Material that may be linked to, or accessed through, an article. The authors have granted ASM a non-exclusive, world-wide license to publish the Supplemental Material files. Please contact the corresponding author directly for reuse.
