# Supplementary material for: Closing the gap: Oxford Nanopore Technologies R10 sequencing allows comparable results to Illumina sequencing for SNP-based outbreak investigation of bacterial pathogens
Source: J Clin Microbiol. 2024 Mar 5;62(5):e01576-23. doi: 10.1128/jcm.01576-23 (PMC11077942; doi:10.1128/jcm.01576-23)
Supplement: Supplemental figures and tables — Fig. S1 to S28 and Tables S1 to S7. [file jcm.01576-23-s0001.docx]

**Supplementary Figures**

## **Figure S1: Read mapping statistics**


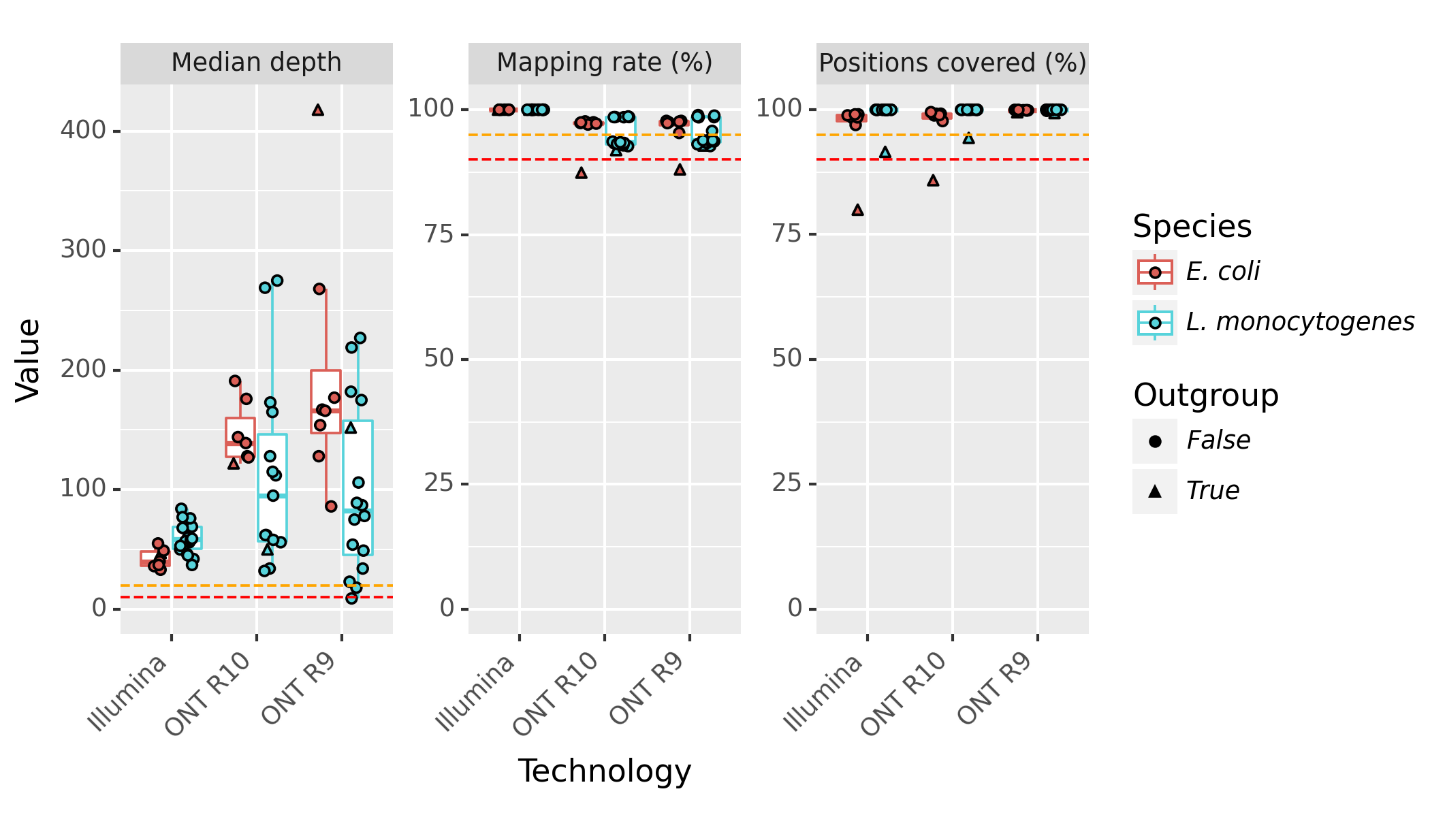


This plot shows the median depth, the mapping rate against the reference genome, and the fraction of the reference genome that is covered. Within each subplot, observations are split by technology and species as indicated on the x-axis. Each points represents an individual dataset and is colored by species. Outgroup isolates are indicated by triangles. The horizontal dashed lines indicate quality thresholds, corresponding to 10x (red) and 20x (orange) median depth, and 90% (red) and 95% (orange) for mapping rate and positions covered.

## **Figure S2: Variant filtering evaluation (depth)**


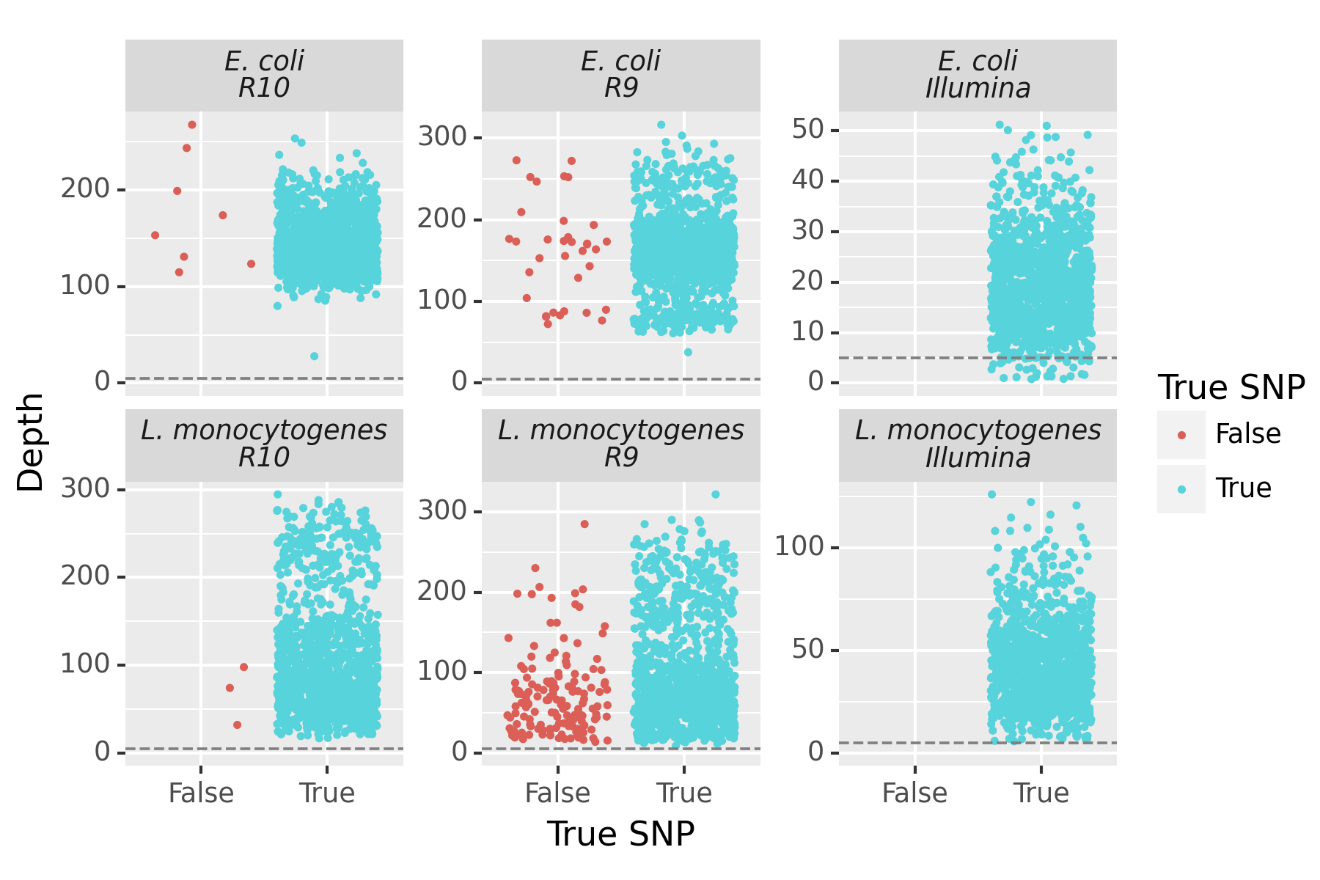


This plot shows the depth values of all SNPs detected in the ONT datasets for both species, extracted from the unfiltered VCF files of the SNP phylogenies containing all three technologies/chemistries. Each point represents a single SNP detected in a dataset. The x-axis shows the two categories of SNPs: true SNPs and false SNPs. The y-axis shows the depth. The horizontal dashed line indicates the threshold value that was used in the workflow. SNPs were considered true SNPs if they were also detected in the corresponding Illumina dataset. Note that the SNPs called in the Illumina data are considered as true SNPs.

## **Figure S3: Variant filtering evaluation (allele frequency)**


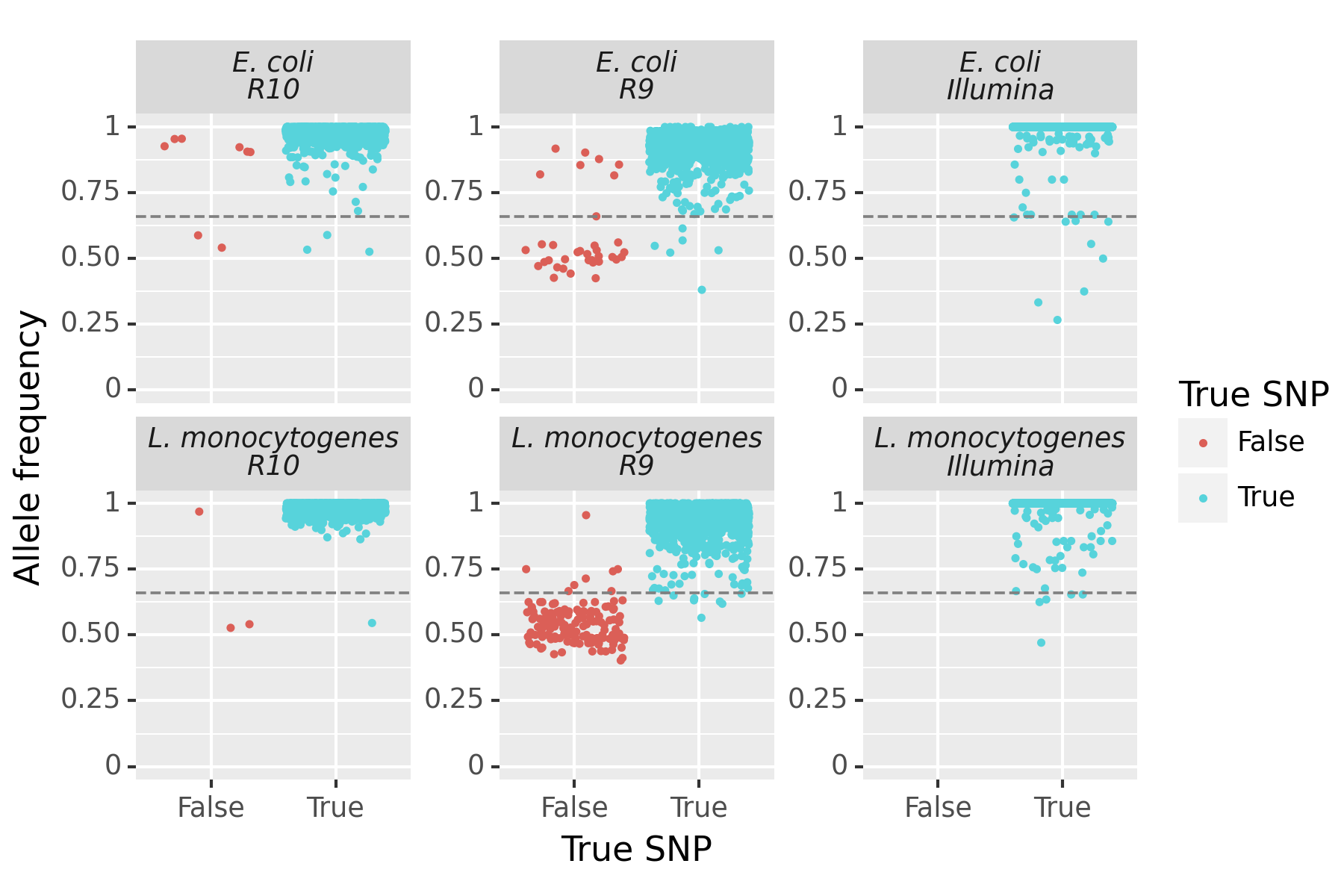


This plot shows the allele frequencies of all SNPs detected in the ONT datasets for both species, extracted from the unfiltered VCF files of the SNP phylogenies containing all three technologies/chemistries. Each point represents a single SNP detected in a dataset. The x-axis shows the two categories of SNPs: true SNPs and false SNPs. The y-axis shows the allele frequency. The horizontal dashed line indicates the threshold value that was used in the workflow (i.e., 66%). SNPs were considered true SNPs if they were also detected in the corresponding Illumina dataset. Note that the SNPs called in the Illumina data are considered true SNPs.

## **Figure S4: Variant filtering evaluation (SNP quality)**


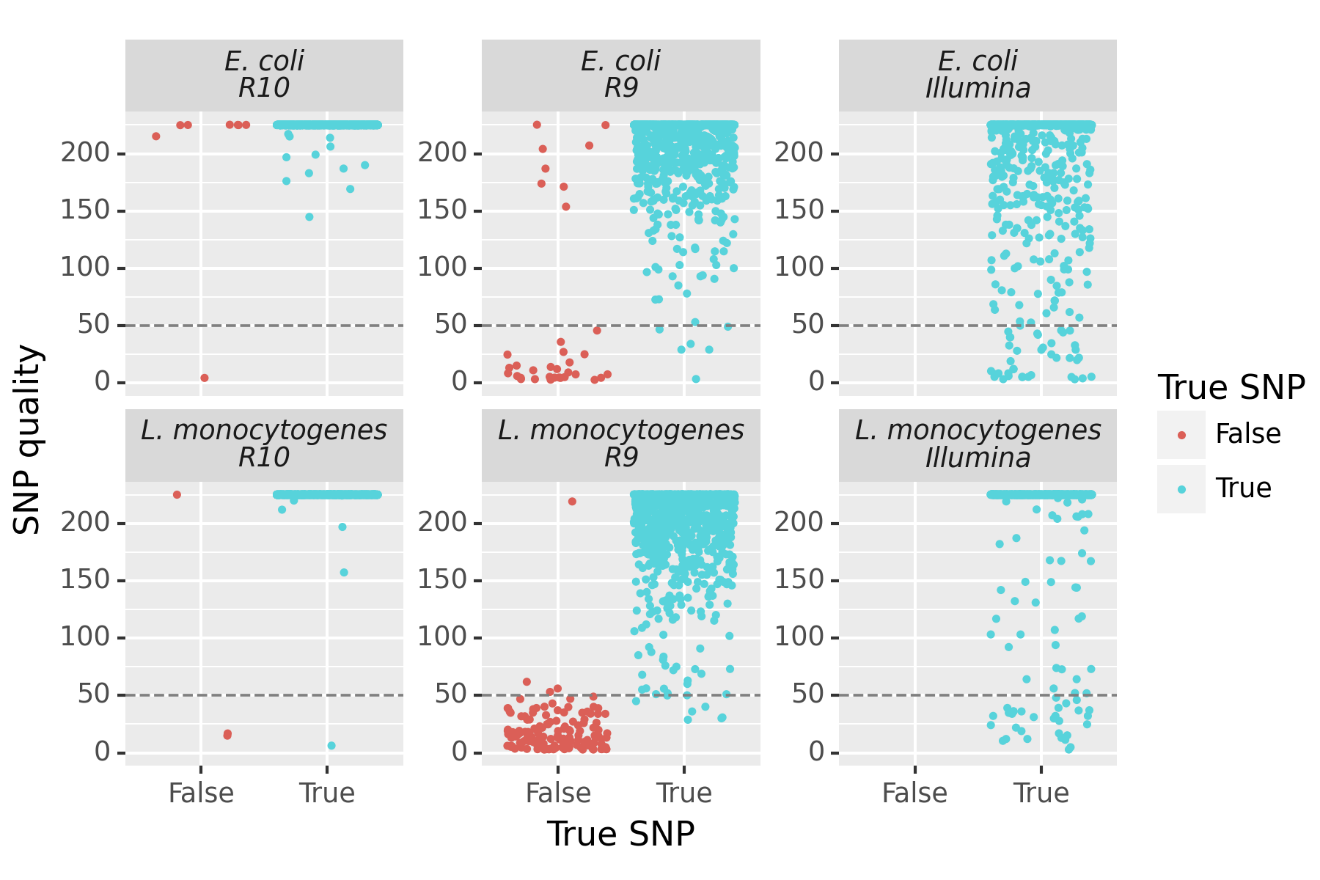


This plot shows the SNP quality of all SNPs detected in the ONT datasets for both species, extracted from the unfiltered VCF files of the SNP phylogenies containing all three technologies/chemistries. Each point represents a single SNP detected in a dataset. The x-axis shows the two categories of SNPs: true SNPs and false SNPs. The y-axis shows the SNP quality. The horizontal dashed line indicates the threshold value that was used in the workflow (i.e., 75). SNPs were considered true SNPs if they were also detected in the corresponding Illumina dataset. Note that the SNPs called in the Illumina data are considered true SNPs.

## **Figure S5: Variant filtering evaluation (P-value strand bias)**


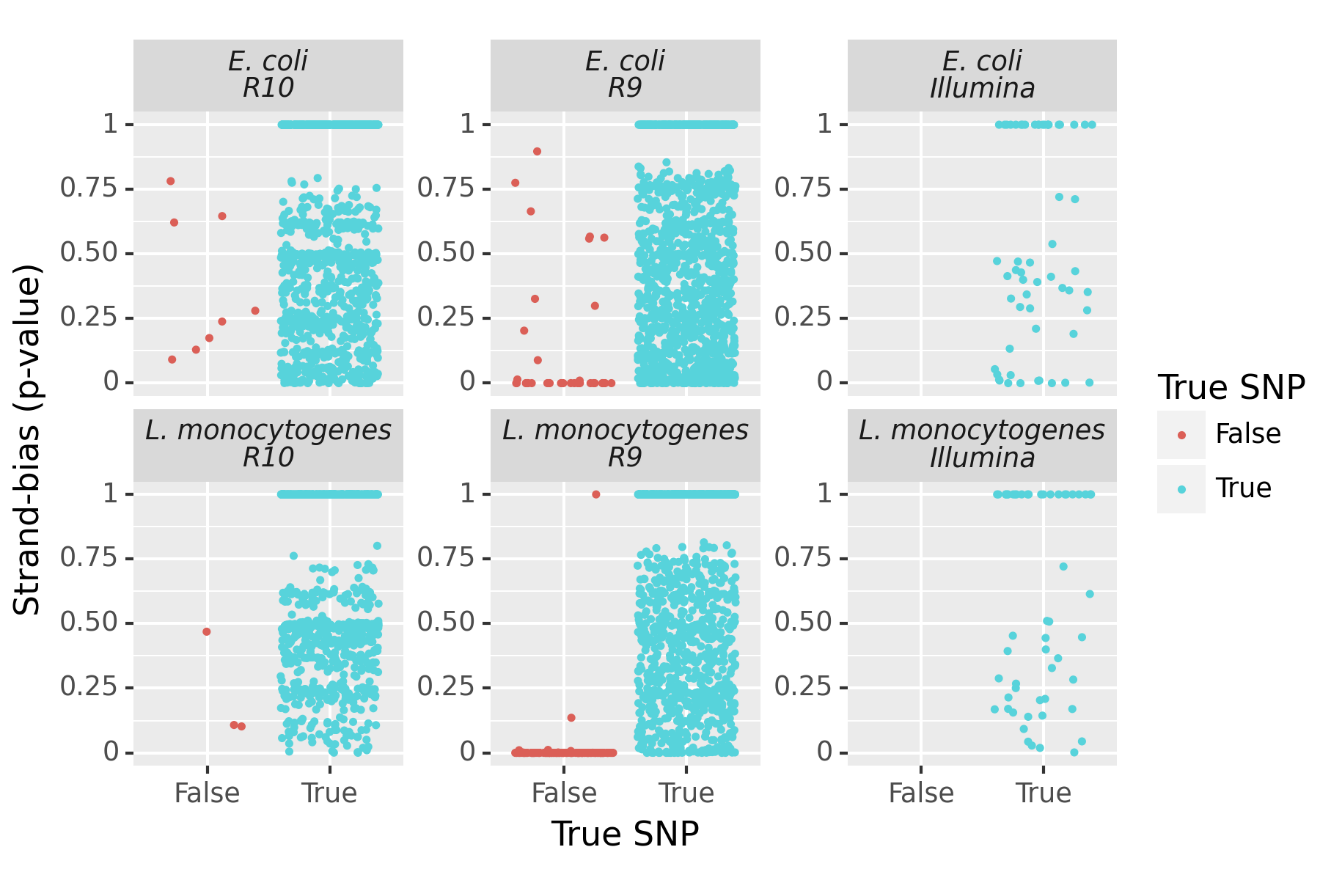


This plot shows the strand bias p-value for all SNPs detected in the ONT datasets for both species, extracted from the unfiltered VCF files of the SNP phylogenies containing all three technologies/chemistries. Each point represents a single SNP detected in a dataset. The x-axis shows the two categories of SNPs: true SNPs and false SNPs. The y-axis shows the strand bias p-value. SNPs were considered true SNPs if they were also detected in the corresponding Illumina dataset. Note that the SNPs called in the Illumina data are considered true SNPs.

## **Figure S6: Mixed position resulting in false SNPs in the *E. coli* datasets**


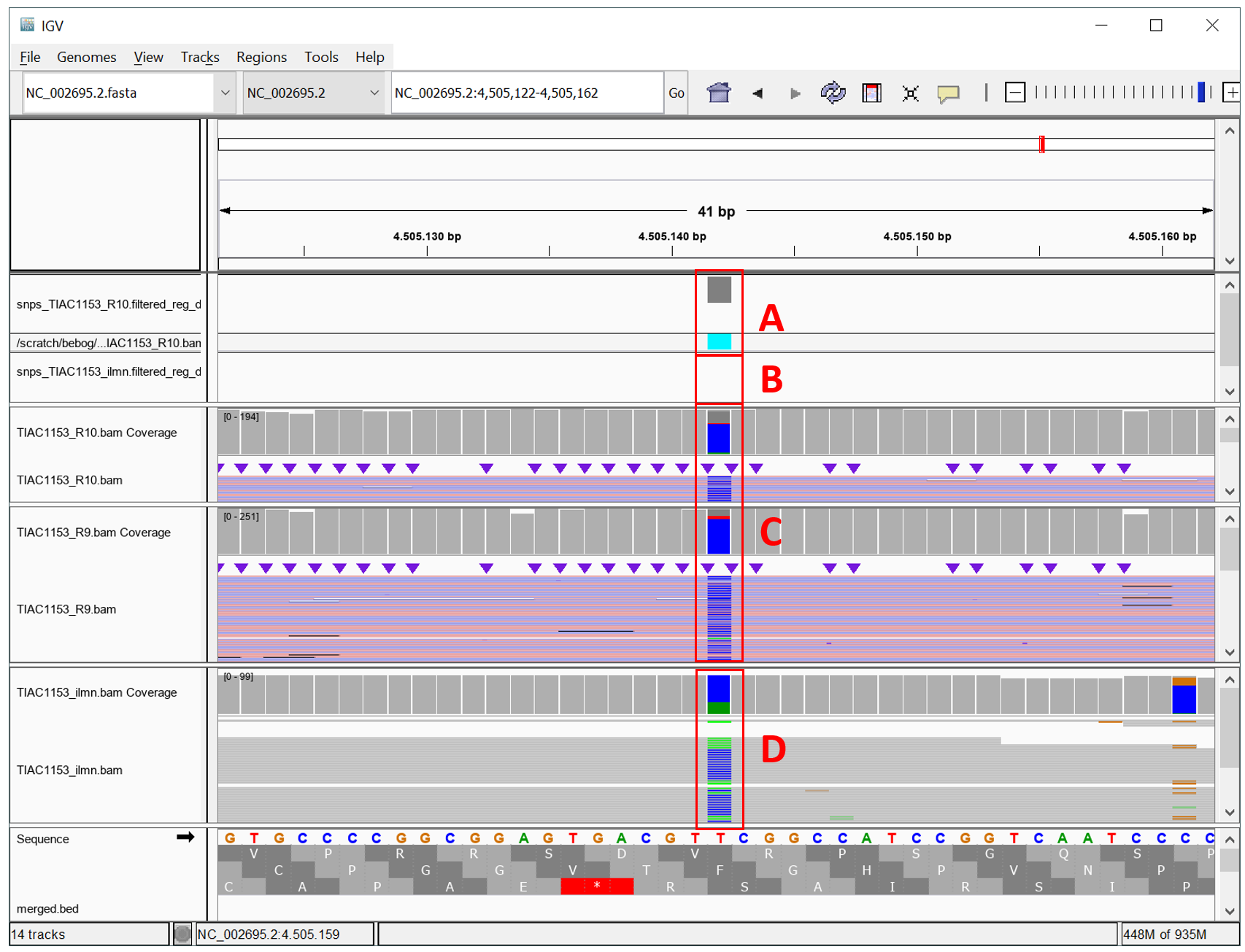


This screenshot shows the alignment around position 4,505,142 in the *E. coli* NC_002695.2 reference genome for the TIAC1153 R10, R9, and Illumina datasets. The letters refer to (A) a SNP detected in the R10 dataset; (B) the absence of a SNP in the Illumina dataset; (C) the presence of ~70% C in the ONT R9 / R10 datasets, with a relatively high fraction of uncalled bases (N), indicated in dark grey; (D) the mixed position in the Illumina data with ~70% C (blue) and ~30% A. This was observed in all *E. coli* datasets, resulting in a total of twelve ‘false’ SNPs (i.e., six R9 and six R10 datasets) with high allele frequencies detected in the ONT R9/R10 datasets.

## **Figure S7: SNPs near recombinant region in the *E. coli* TIAC1153 dataset**

**
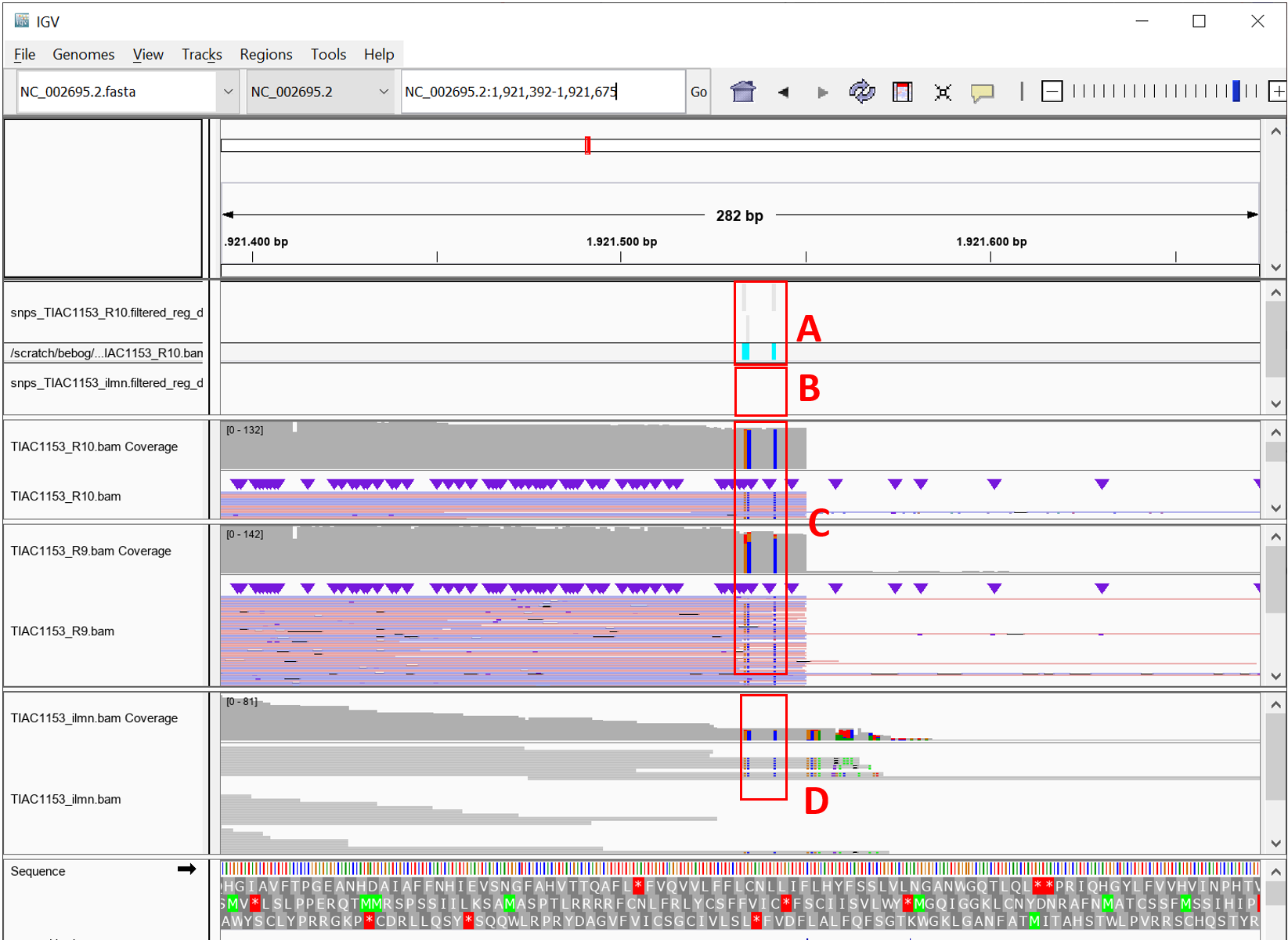
**

This plot shows the alignment around position 1,921,534 in the *E. coli* NC_002695.2 reference genome for the TIAC1153 R10, R9, and Illumina datasets. The letters refer to (A) three SNPs detected in the R10 dataset; (B) the absence of SNPs in the Illumina dataset; (C) the reads supporting the SNPs in the ONT R9 / R10 datasets; (D) the reads supporting the SNPs in the Illumina data, with numerous additional variants on the right side indicating recombination. In total, eight of these ‘false’ SNPs (i.e., four in the R9 and four in the R10 datasets) were detected in the TIAC1153 datasets. Note that the variants detected in the TIAC1153 ONT datasets were filtered out by the SNP distance filter, as they were located within 10 bp of each other. This phenomenon was not observed for the other isolates, which were more closely related to the reference genome. This region was not filtered out by Gubbins as this variation was unique to this particular isolate.

## **Figure S8: SNP calling and filtering statistics**


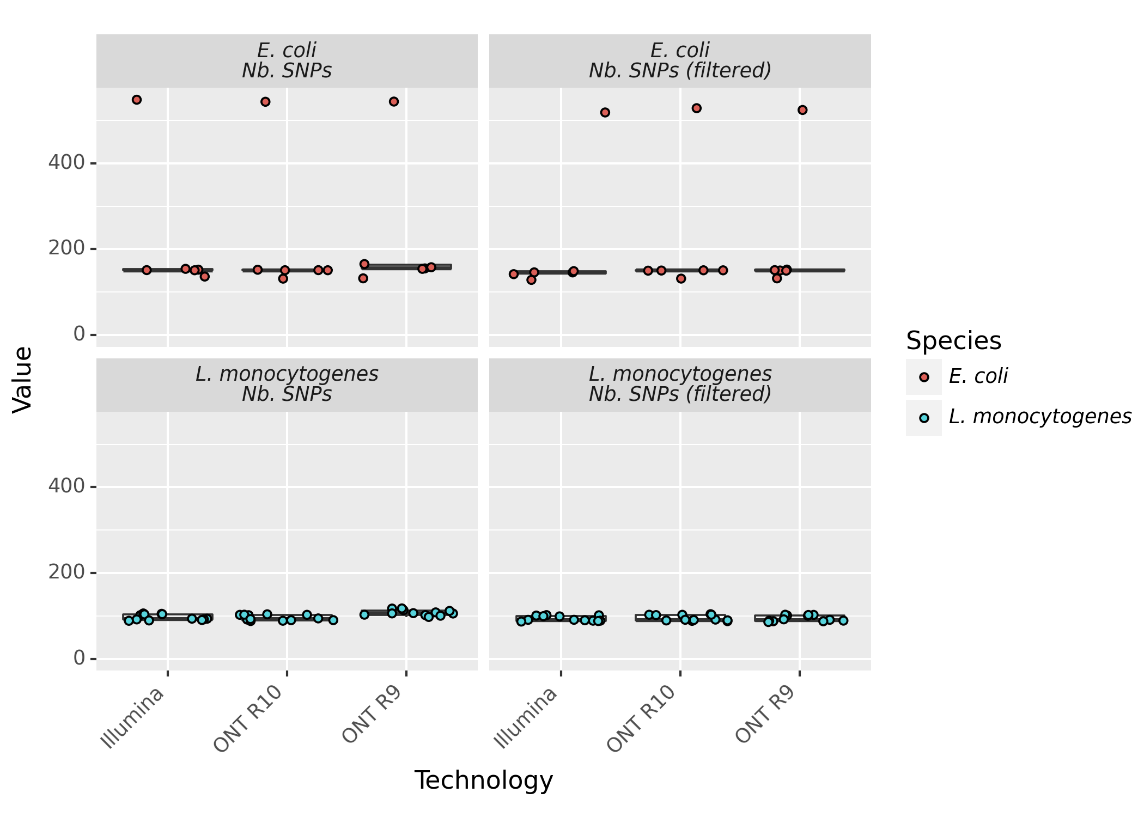


This plot shows the number of SNPs before and after filtering for the *E. coli* and *L. monocytogenes* datasets. The outgroup isolates were not included in this plot. For *E. coli*, the higher number of SNPs corresponds to isolate TIAC1153, which is quite distant from the outbreak isolates and the reference genome (see Figure 1). Note that these values were calculated based on the SNPs called in regions that passed region filtering, as detailed in Section 2.6.

## **Figure S9: Comparison between *L. monocytogenes* phylogenies generated with Illumina, R9, and R10 data**

| **Phylogeny** | | | |
| --- | --- | --- | --- |
| **Illumina** | **R10** | | **R9** |
| 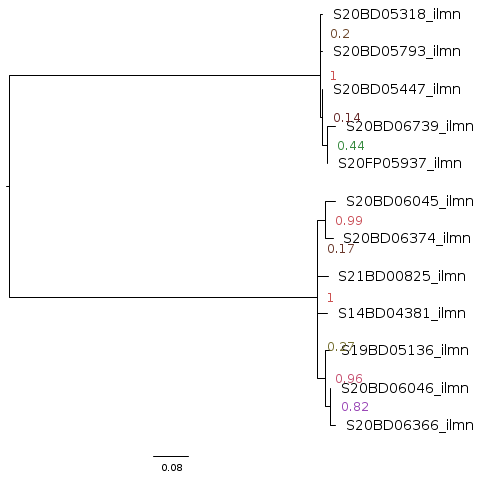 | 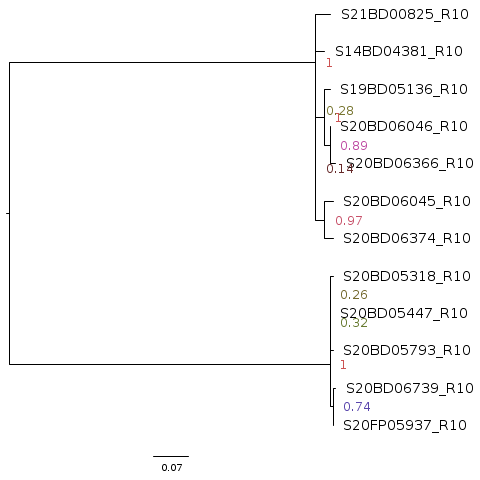 | | 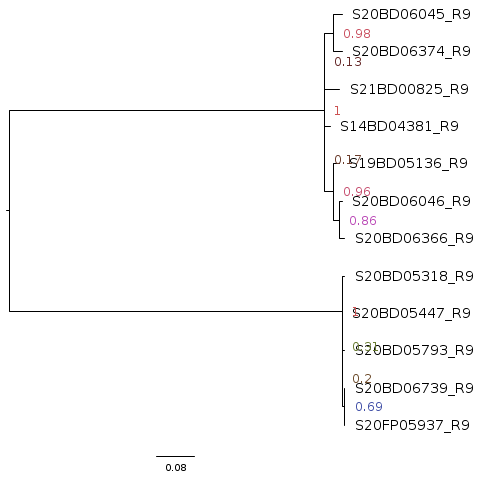 |
| **Pairwise SNP matrix** | | | |
| **Illumina** | | **R10** | **R9** |
| 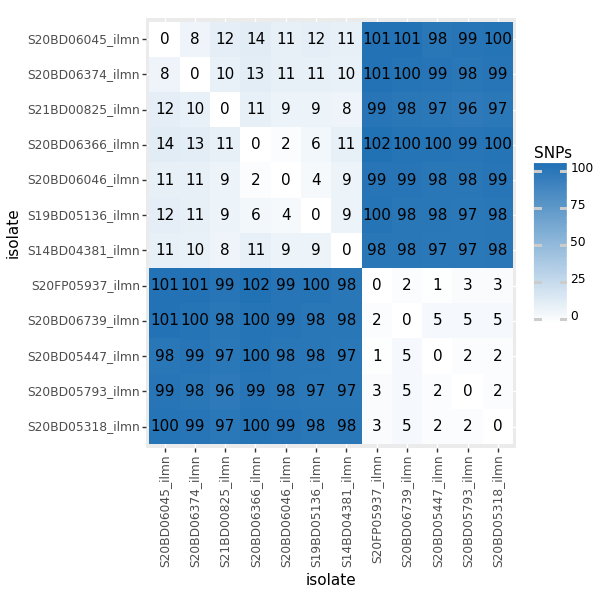 | | 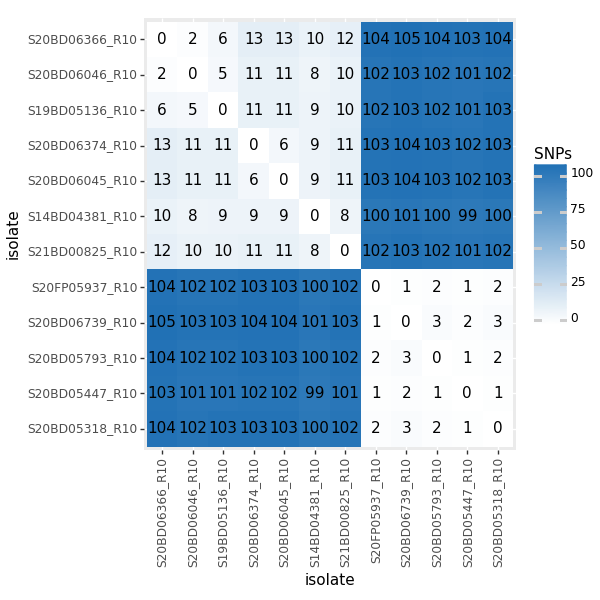 | 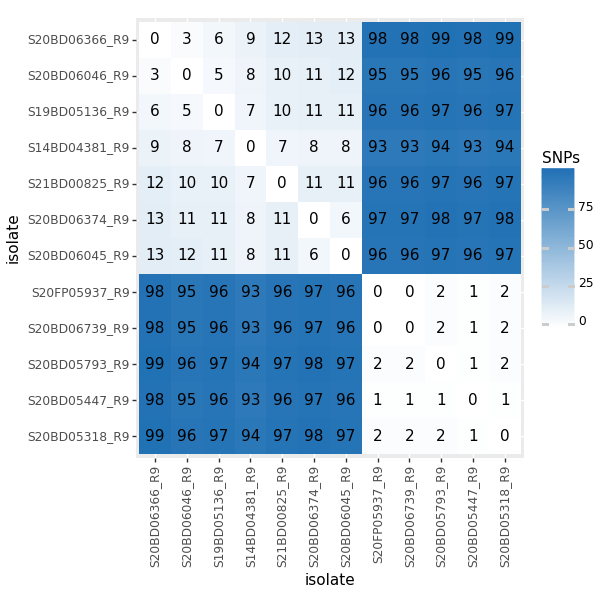 |

## **Figure S10: Region filtering for the SNP phylogenies**


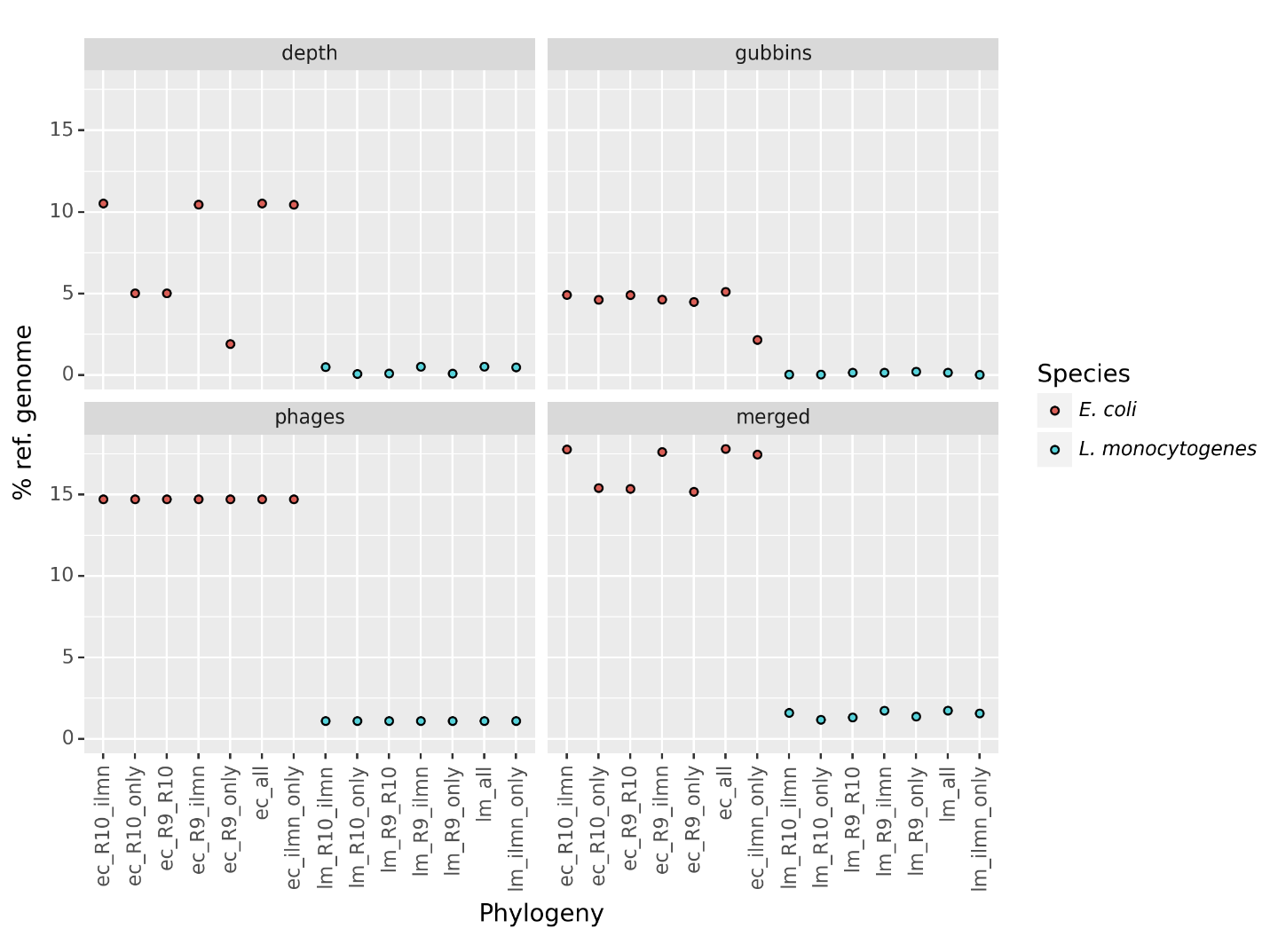


These plots show the percentage of the reference genome (y-axis) that was omitted from the SNP analysis for the different phylogenies (x-axis). The y-axis shows the percentage of the reference genome that is covered by each category. The subplots show the regions below the 5x depth cutoff (‘depth’), regions marked as recombinant by Gubbins (‘gubbins’), regions identified as (pro-)phages (‘phages’), and the merged BED file combining the three others (‘merged’). The points are colored by species as indicated in the legend.

## **Figure S11: SNP matrix statistics**


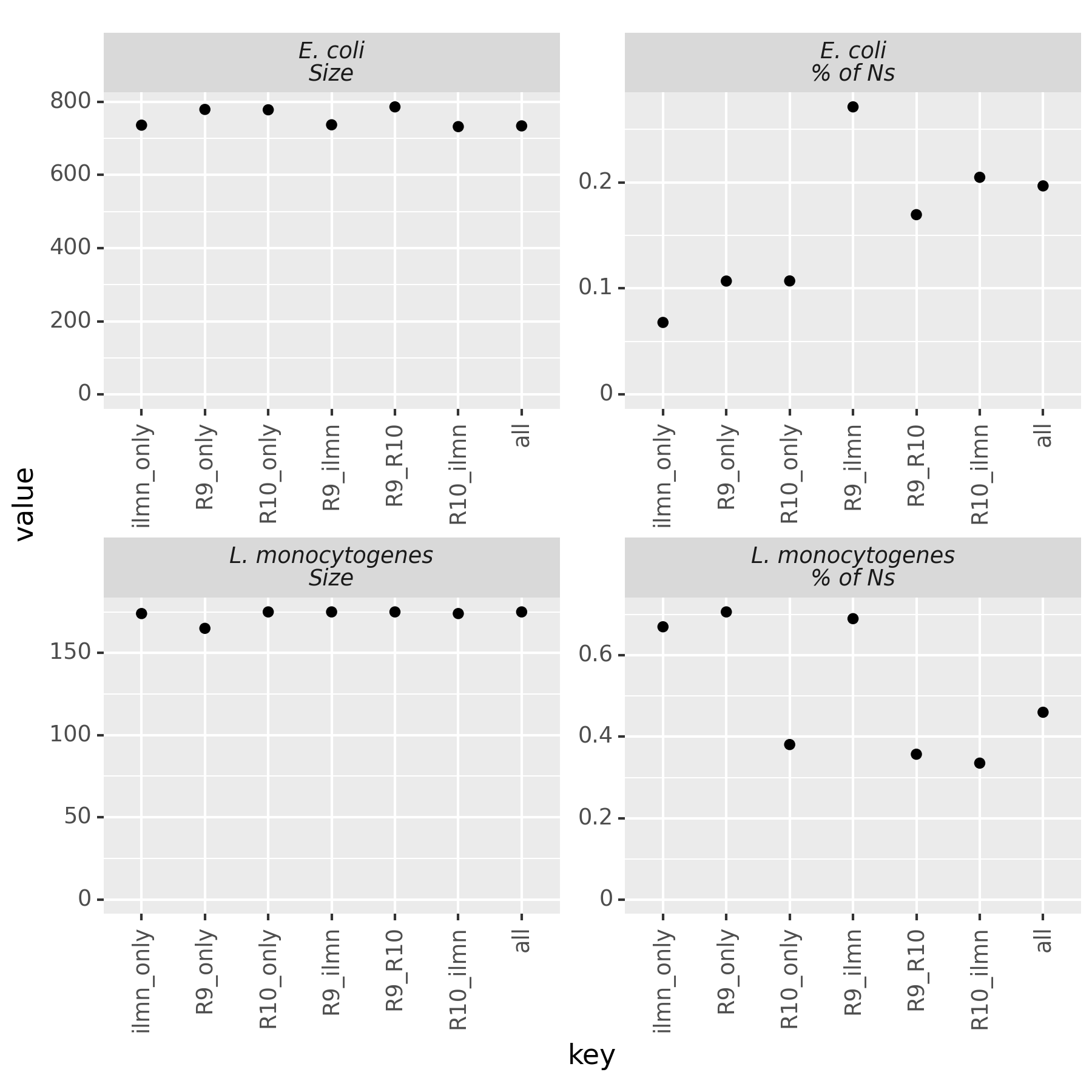


These plots show the statistics for the SNP matrices. The x-axis indicates the sequencing technologies that were included in the phylogeny. The mixed phylogenies correspond to the phylogenies containing all isolates sequenced with the corresponding technologies/chemistries. The y-axis indicates the value of the corresponding metric. Note that the scale is different for each subplot. Abbreviations: Illumina (ilmn).

## **Figure S12: Distances between the phylogenetic groups of the *E. coli* phylogeny**


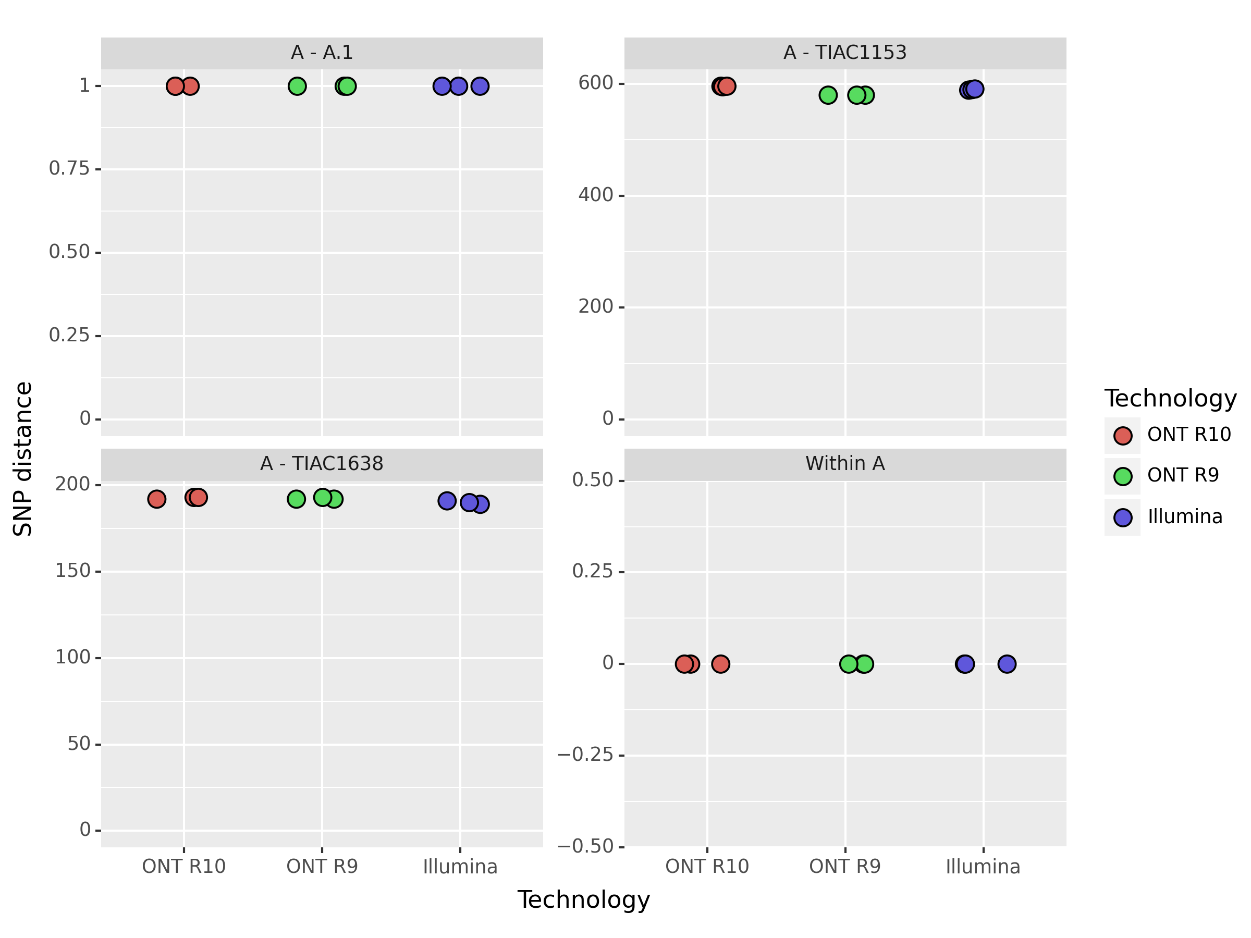


These plots show the pairwise SNP distances between different groups and/or isolates in the *E. coli* phylogenies. The group compositions are shown in Figure 1a. Each point represents a comparison between two isolates and is colored by sequencing technology as indicated in the legend (and on the x-axis). SNP distances were extracted from the phylogenies containing only the datasets generated by the respective technology.

## **Figure S13: Distances between the phylogenetic groups of the *L. monocytogenes* phylogeny**


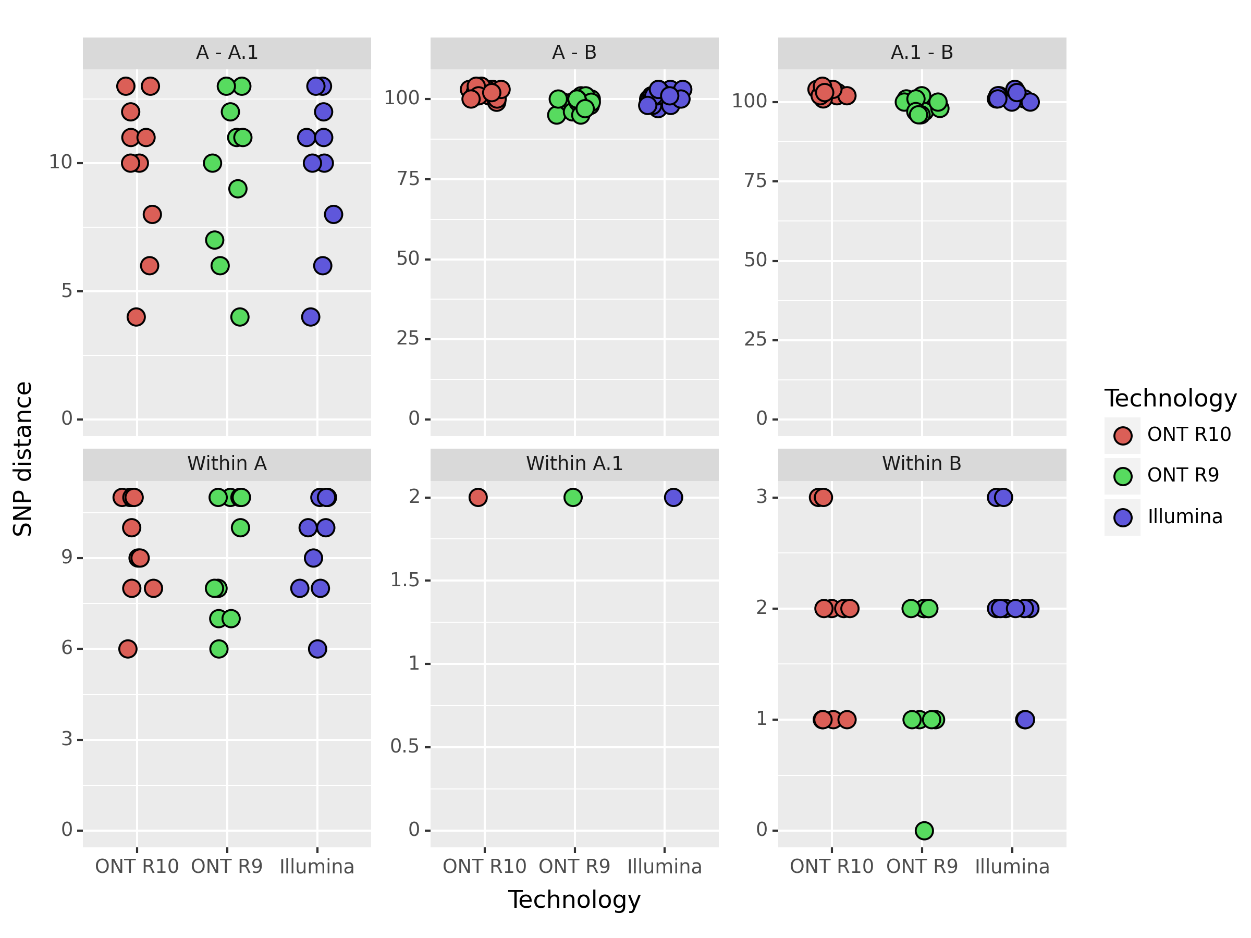


These plots show the pairwise SNP distances between different groups and/or isolates in the *L. monocytogenes* phylogenies. The group compositions are shown in Figure 1b. Each point represents a comparison between two isolates and is colored by sequencing technology as indicated in the legend (and on the x-axis). SNP distances were extracted from the phylogenies containing only the datasets generated by the respective technology.

## **Figure S14: Combined phylogeny of the *E. coli* Illumina and R9 datasets**

| **Phylogeny** |
| --- |
| 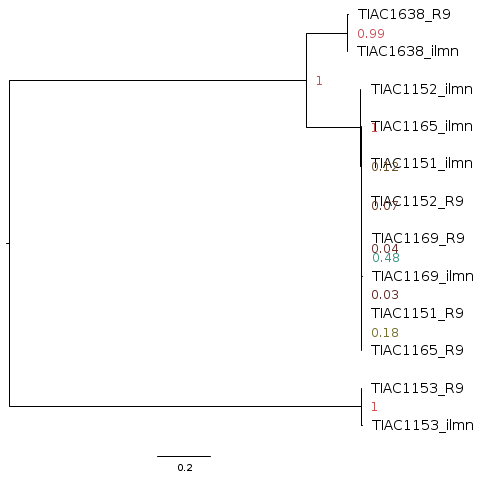 |
| **Pairwise SNP matrix** |
| **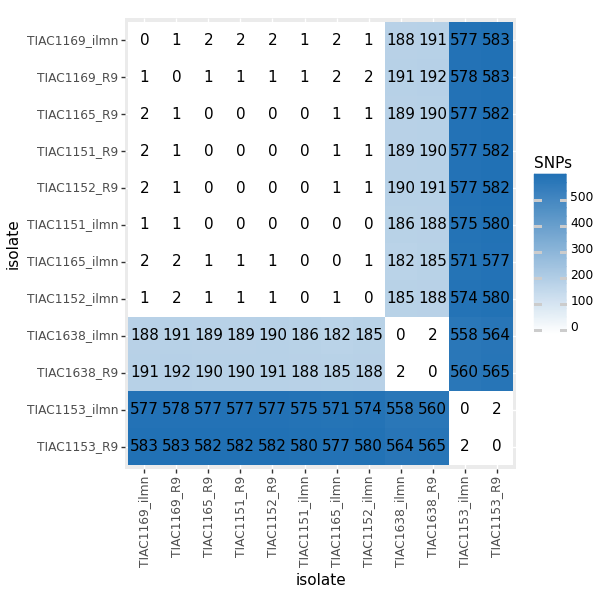** |

This figure shows the results of the phylogenetic analysis for the *E. coli* Illumina datasets combined with the R9 datasets, including the phylogenetic tree (top) and the pairwise SNP distances (bottom).

## **Figure S15: Combined phylogeny of the *E. coli* Illumina and R10 datasets**

| **Phylogeny** |
| --- |
| 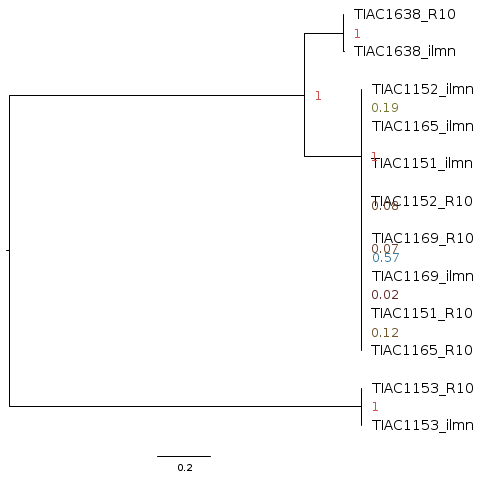 |
| **Pairwise SNP matrix** |
| **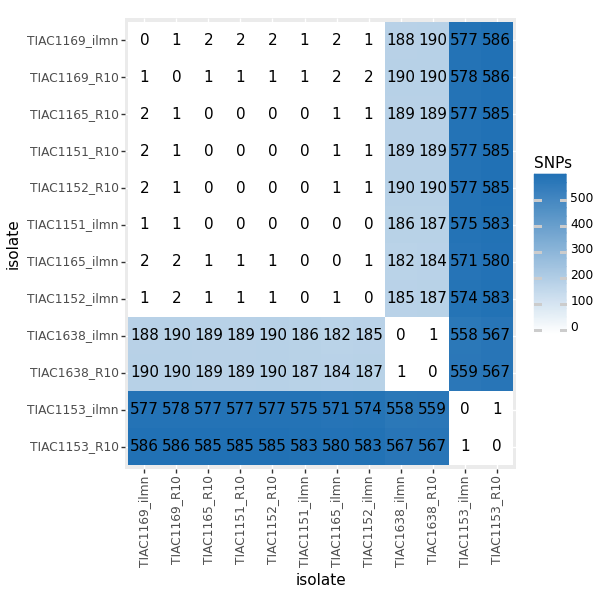** |

This figure shows the results of the phylogenetic analysis for the *E. coli* Illumina datasets combined with the R10 datasets, including the phylogenetic tree (top) and the pairwise SNP distances (bottom).

## **Figure S16: Combined phylogeny of the *E. coli* R9 and R10 datasets**

| **Phylogeny** |
| --- |
| 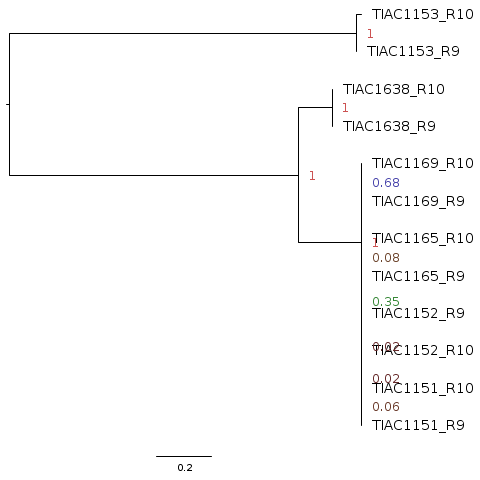 |
| **Pairwise SNP matrix** |
| **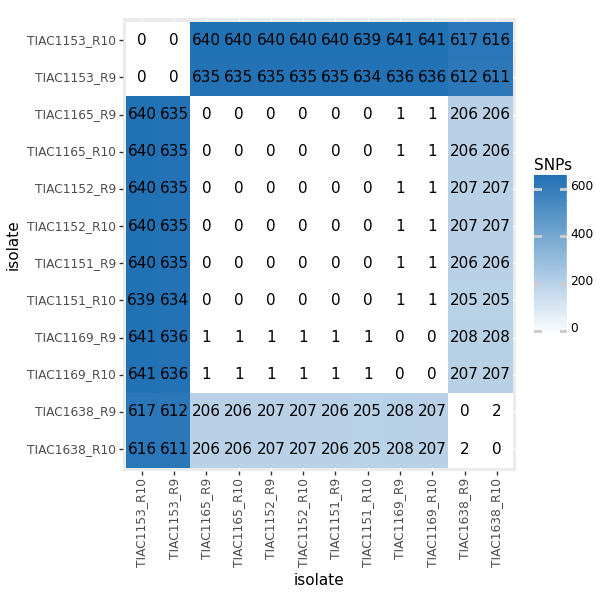** |

This figure shows the results of the phylogenetic analysis for the *E. coli* R9 datasets combined with the R10 datasets, including the phylogenetic tree (top) and the pairwise SNP distances (bottom).

## **Figure S17: Combined phylogeny of the *E. coli* Illumina, R9 and R10 datasets**

| **Phylogeny** |
| --- |
| 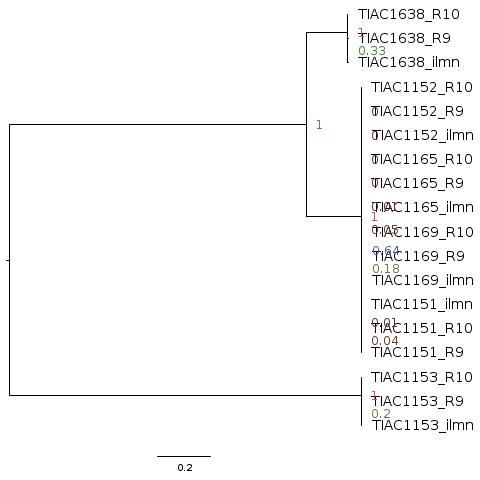 |
| **Pairwise SNP matrix** |
| **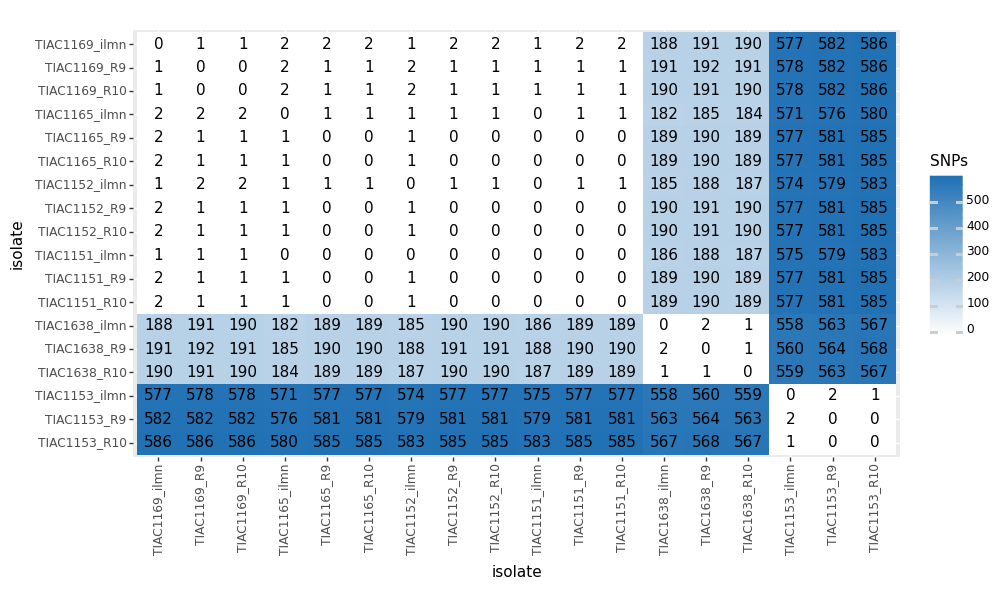** |

This figure shows the results of the phylogenetic analysis for the *E. coli* R9 datasets combined with the R10 datasets, including the phylogenetic tree (top) and the pairwise SNP distances (bottom).

## **Figure S18: Combined phylogeny of the *L. monocytogenes* Illumina and R9 datasets**

| **Phylogeny** |
| --- |
| 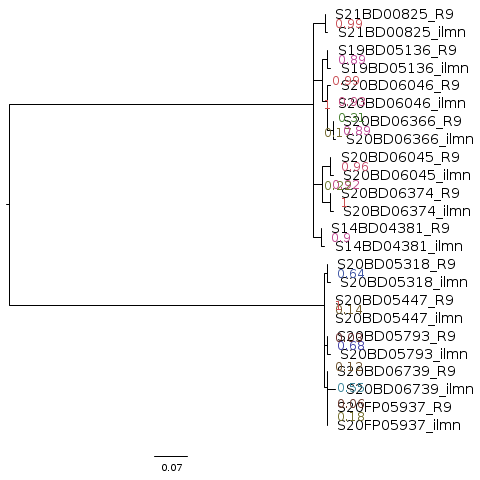 |
| **Pairwise SNP matrix** |
| **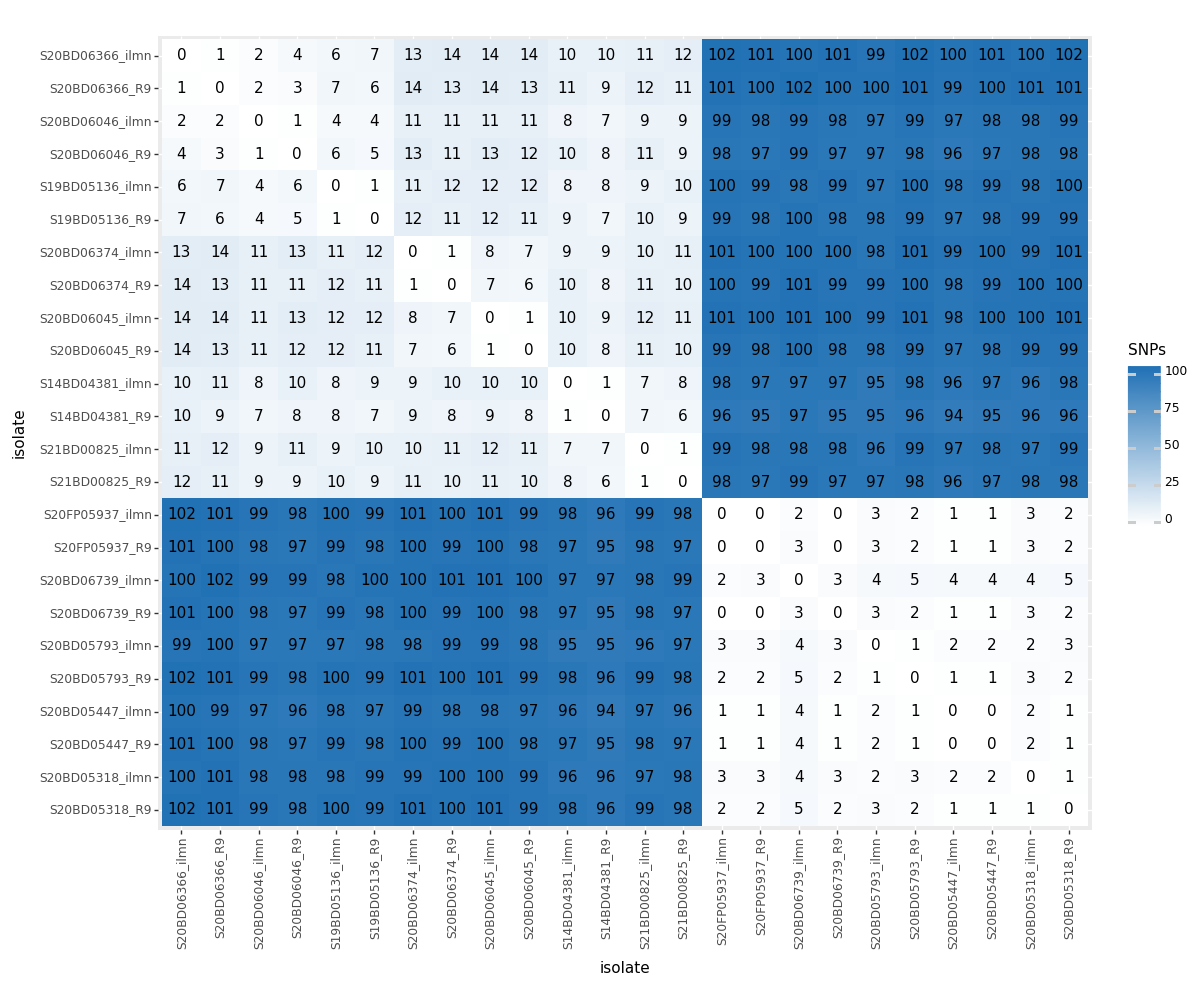** |

This figure shows the results of the phylogenetic analysis for the *E. coli* Illumina datasets combined with the R9 datasets, including the phylogenetic tree (top) and the pairwise SNP distances (bottom).

## **Figure S19: Combined phylogeny of the *L. monocytogenes* Illumina and R10 datasets**

| **Phylogeny** |
| --- |
| 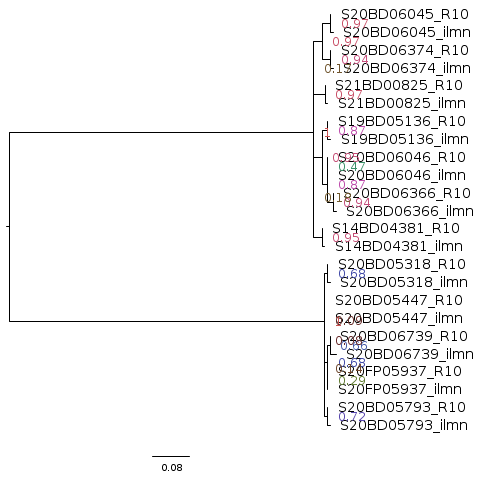 |
| **Pairwise SNP matrix** |
| **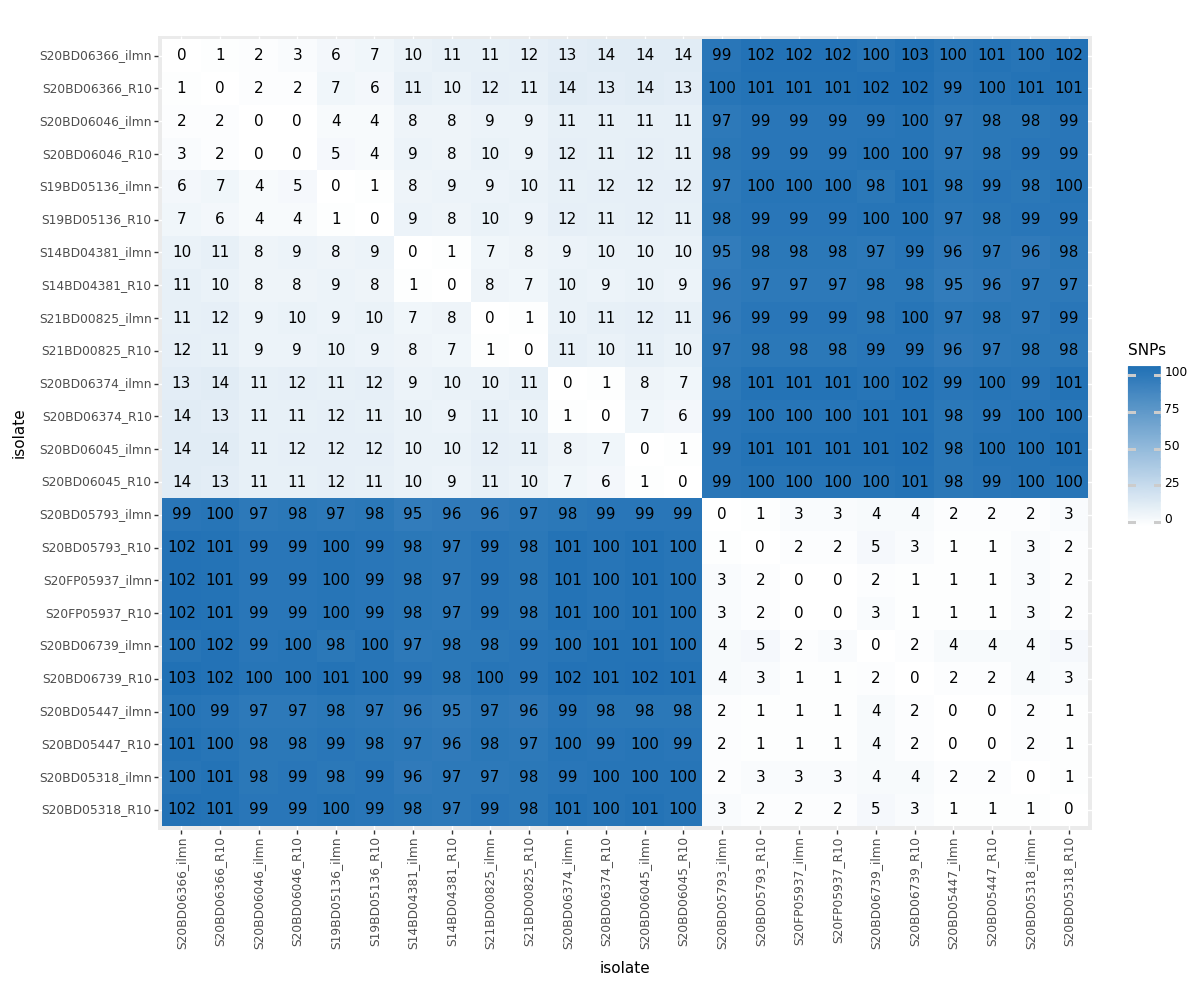** |

This figure shows the results of the phylogenetic analysis for the *E. coli* Illumina datasets combined with the R10 datasets, including the phylogenetic tree (top) and the pairwise SNP distances (bottom).

## **Figure S20: Combined phylogeny of the *L. monocytogenes* R9 and R10 datasets**

| **Phylogeny** |
| --- |
| 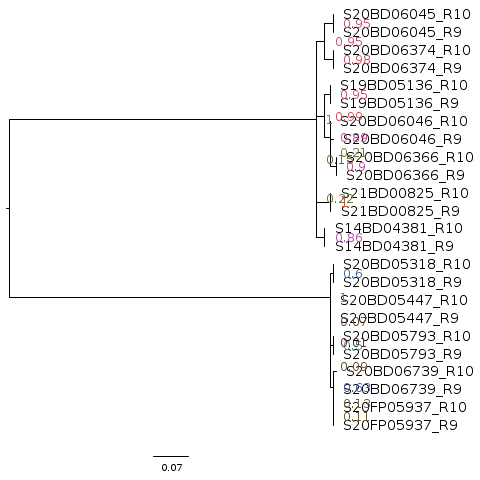 |
| **Pairwise SNP matrix** |
| **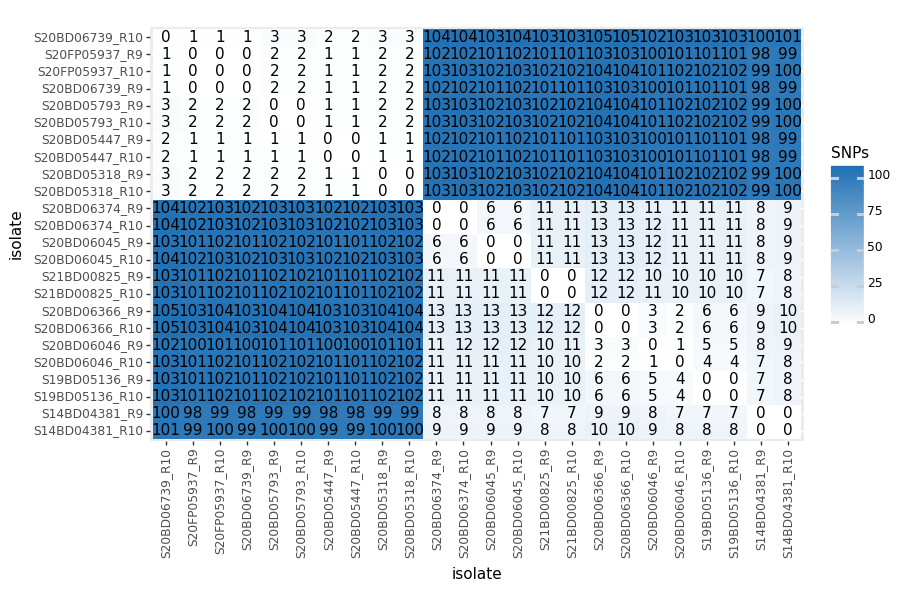** |

This figure shows the results of the phylogenetic analysis for the *E. coli* R9 datasets combined with the R10 datasets, including the phylogenetic tree (top) and the pairwise SNP distances (bottom).

## **Figure S21: Combined phylogeny of the *L. monocytogenes* Illumina, R9 and R10 datasets**

| **Phylogeny** |
| --- |
| 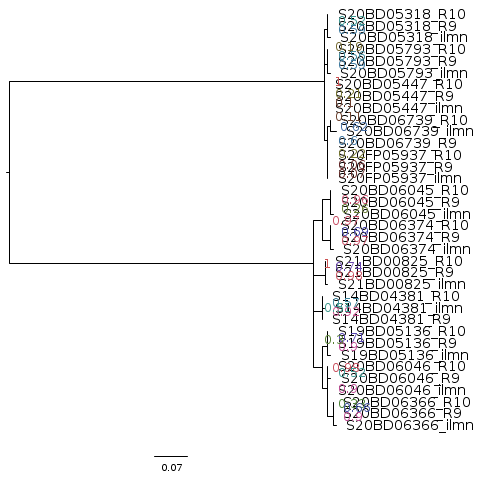 |
| **Pairwise SNP matrix** |
| **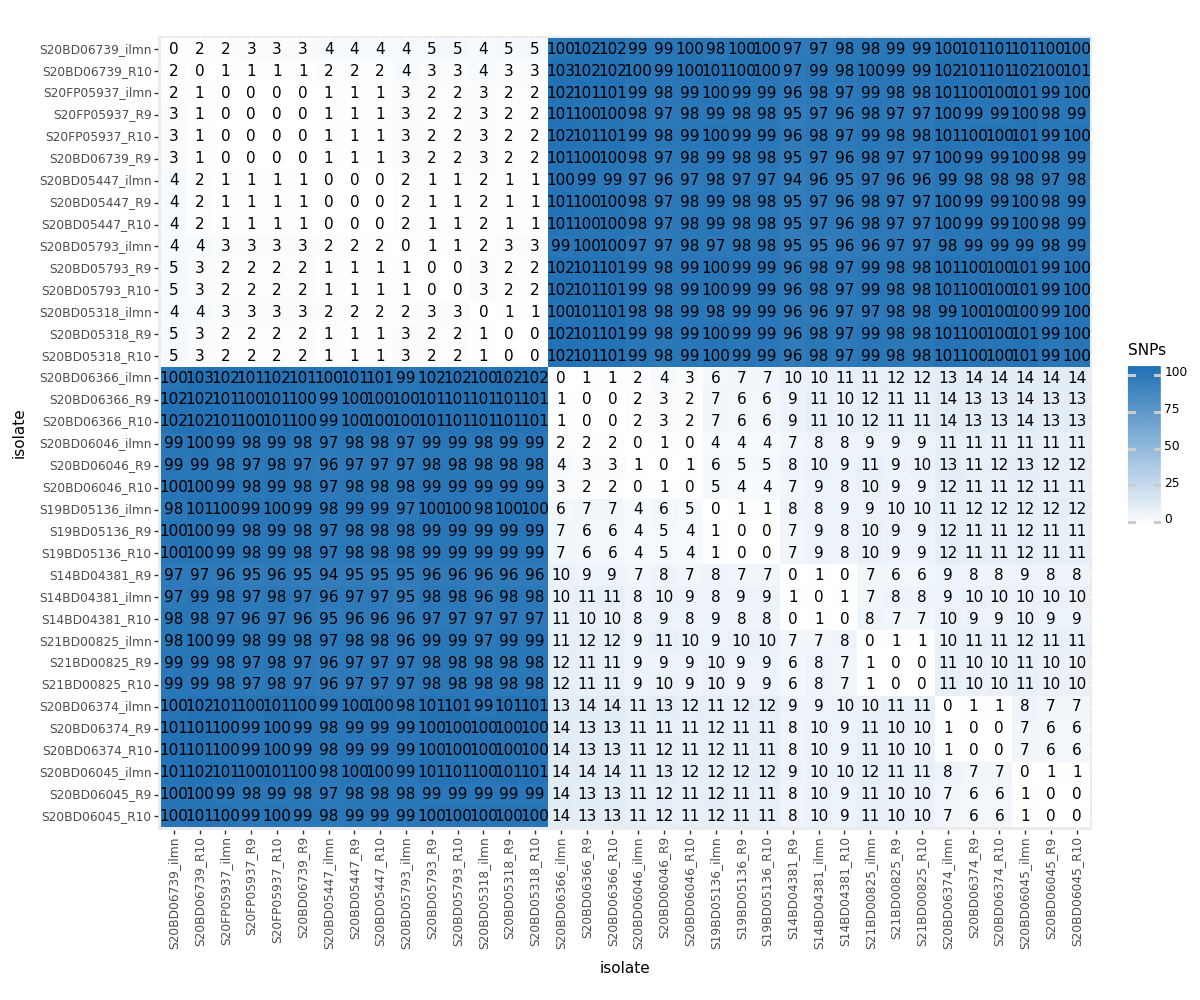** |

This figure shows the results of the phylogenetic analysis for the *E. coli* R9 datasets combined with the R10 datasets, including the phylogenetic tree (top) and the pairwise SNP distances (bottom).

## **Figure S22: Benchmarking of the complete SNP phylogeny analysis**


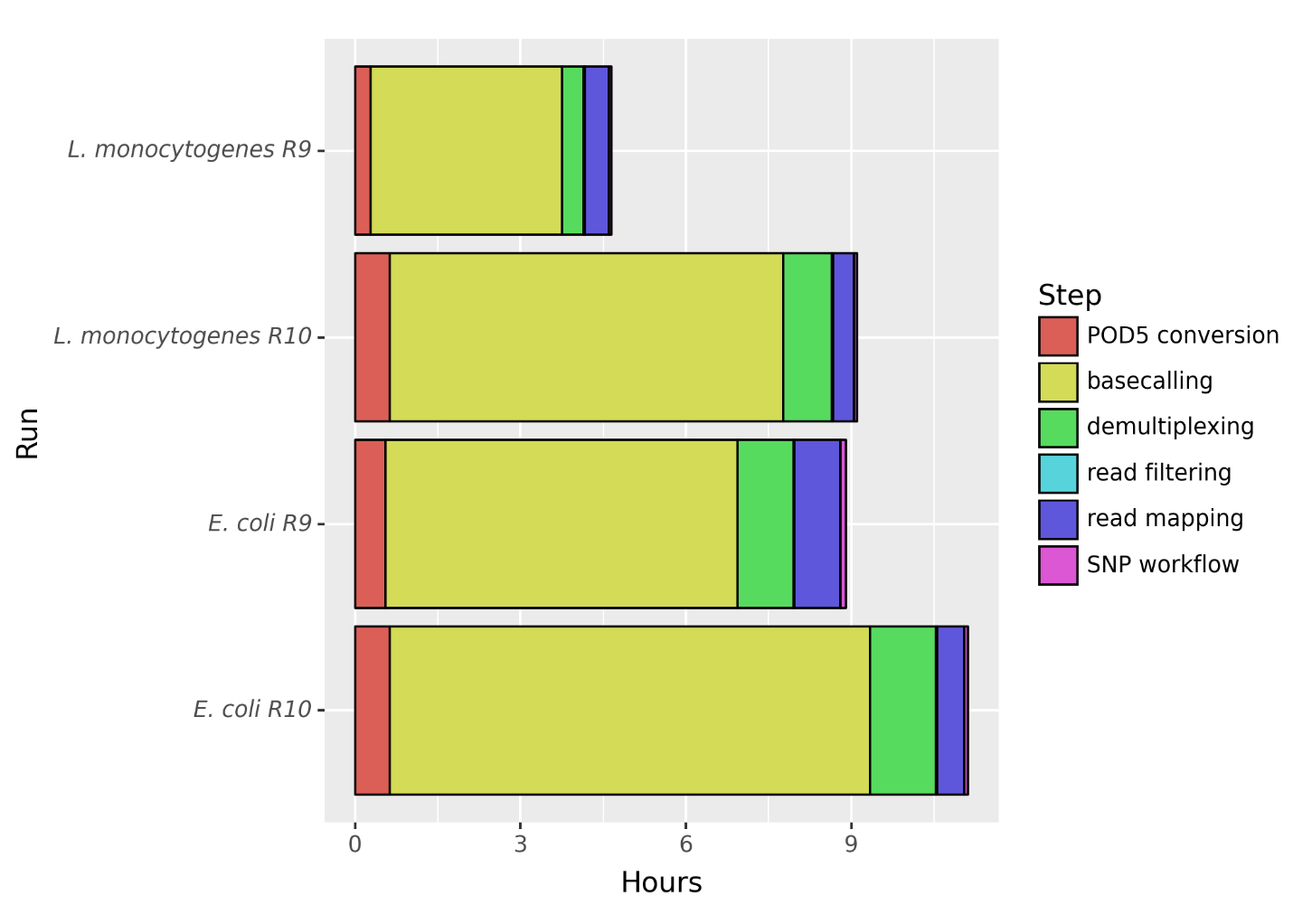


This graph shows the duration of the steps in the SNP phylogeny analysis. The x-axis represents the processing time in hours. The y-axis represents the four runs. Note that the time to generate the PHASTER phage BED file was not included. Note also that the *L. monocytogenes* R9 run had a considerably lower yield than the other three runs, which most likely explains the much shorter run time. The ‘read mapping’ step refers to phase 1 of the PACU workflow shown in Figure 2 (i.e., mapping the reads to the reference genome). The ‘SNP workflow’ step refers to phases 2 to 4 (i.e., variant calling, variant filtering, and phylogenetic tree construction).

## **Figure S23: Reads sequenced in function of sequencing time**

*
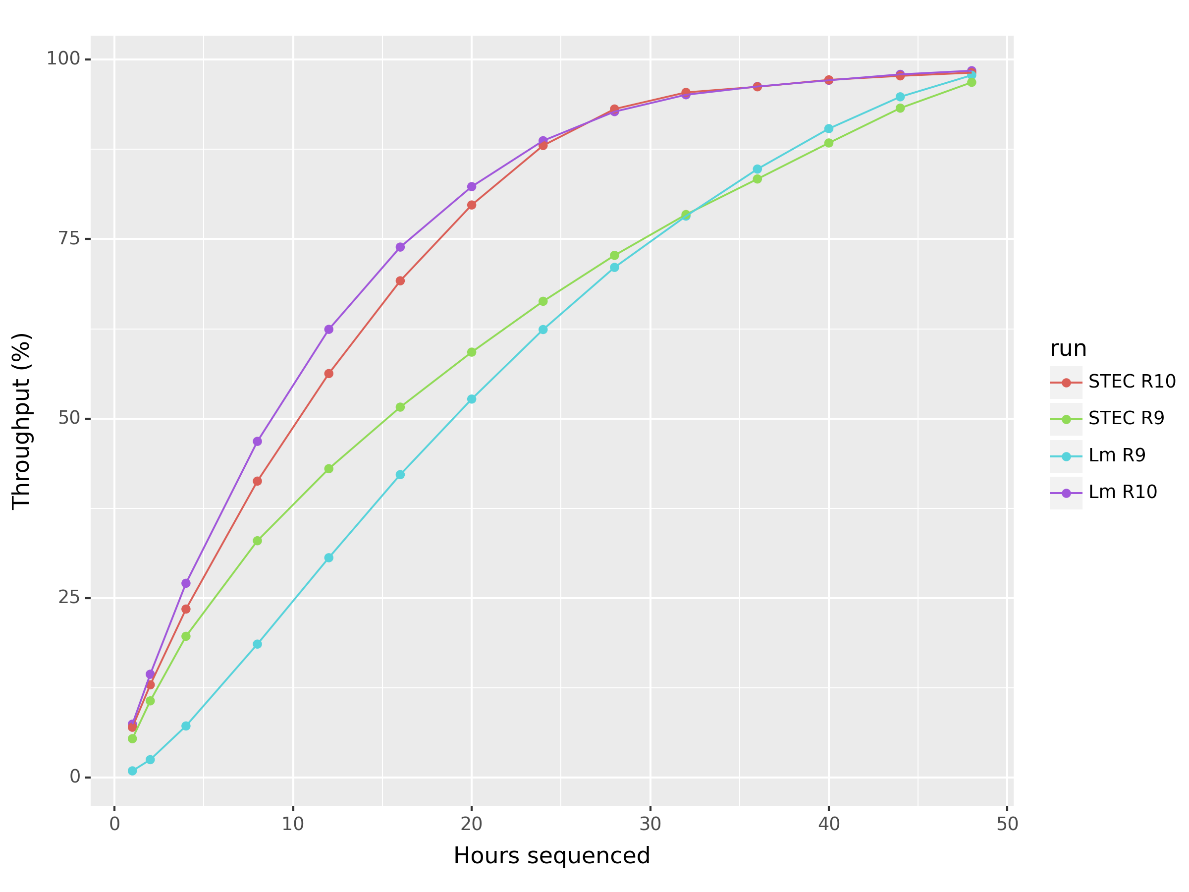
*

The x-axis represents the number of hours sequenced. The Y-axis represents the throughput, calculated as the number of reads sequenced at the corresponding time point divided by the total number of reads. Note that this throughput calculation does not consider read length variation. Abbreviations: *Listeria monocytogenes* (Lm).

## **Figure S24: Datasets suitable for SNP analysis in function of sequencing time**

*
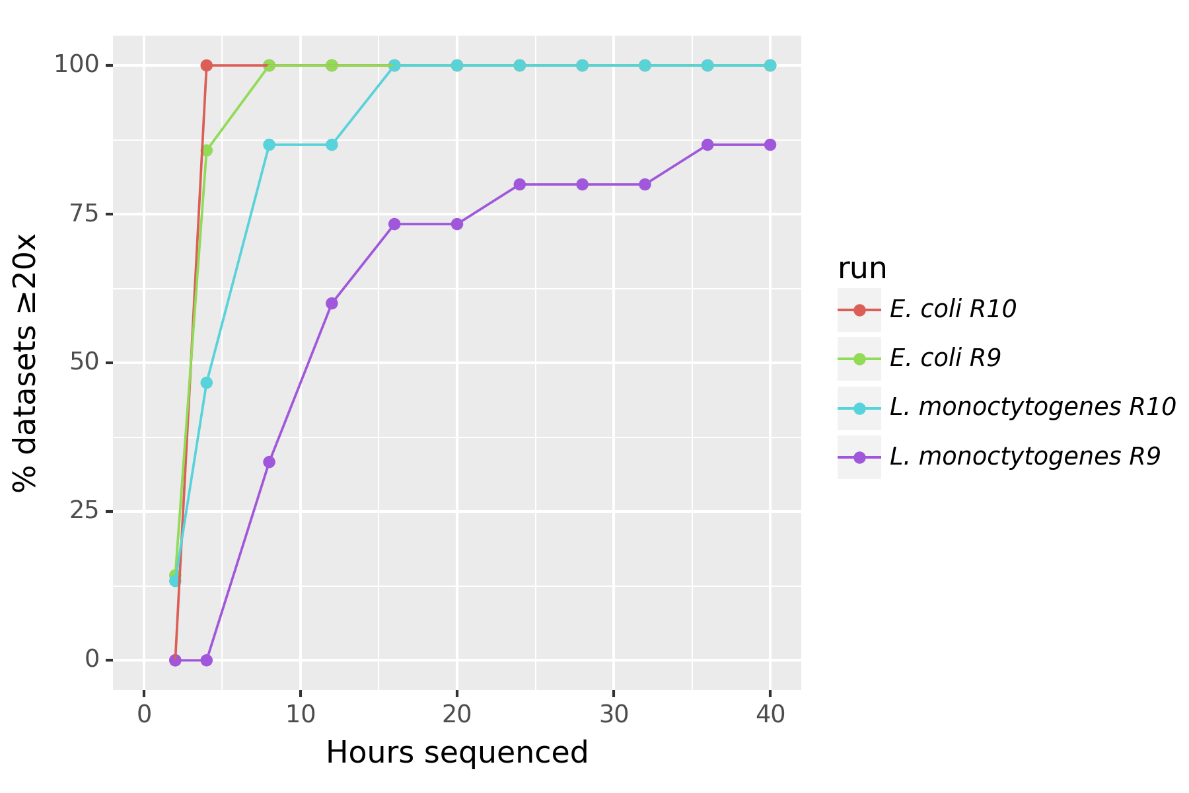
*

The x-axis represents the number of hours sequenced. The y-axis represents the percentage of datasets above the 20x depth cutoff, indicating that they were suitable for SNP analysis. For the *L. monocytogenes* R9 run, only 13 of the 15 isolates were above the threshold after sequencing.

## **Figure S25: Kendall-Colijn distance of the phylogeny at different hours sequenced**

*
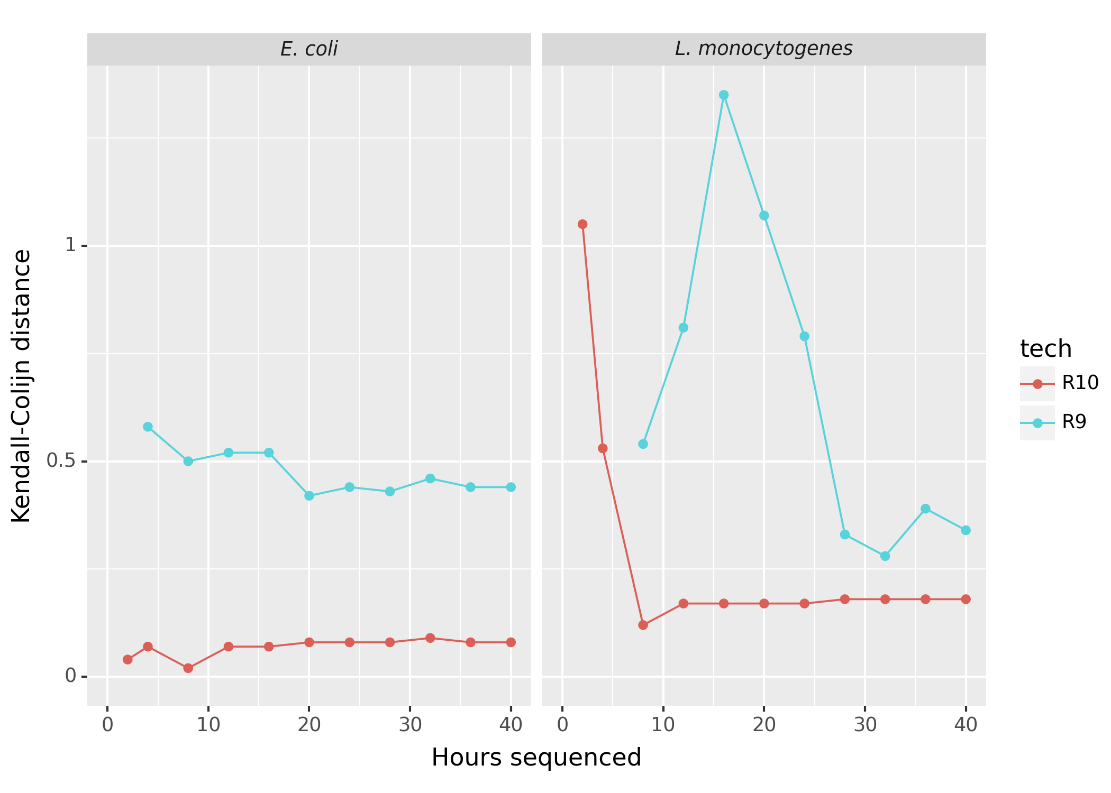
*

The x-axis represents the number of hours sequenced. The y-axis shows the Kendall-Colijn distance of the phylogeny generated at the corresponding sequencing time to the Illumina reference phylogeny. For the R9 datasets, tree construction failed due to insufficient data for the two-hour *E. coli* phylogeny and the two-hour and four-hour *L. monocytogenes* phylogenies.

## **Figure S26: Kendall-Colijn distance of the phylogeny at different number of reads**


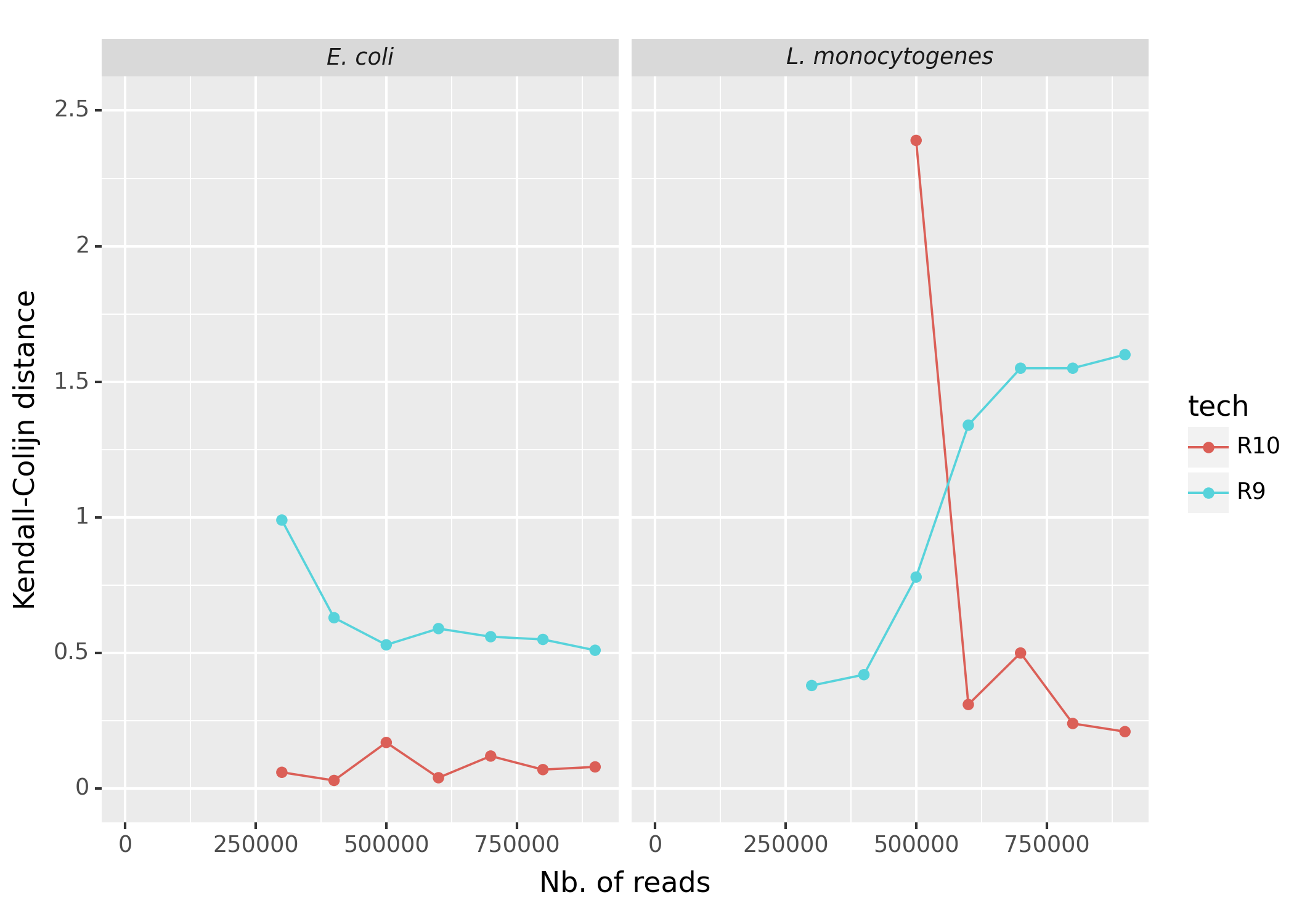


The x-axis represents the number of reads sequenced. The y-axis shows the Kendall-Colijn distance of the phylogeny generated at the corresponding sequencing time to the Illumina reference phylogeny. Data points for which the tree construction failed or for which the Kendall-Colijn distance was larger than 10 were not shown. Note that the R9 reads tended to be longer than the R10 reads.

## **Figure S27: Core-genome MLST (cgMLST) phylogeny for hybrid assemblies for *E. coli***


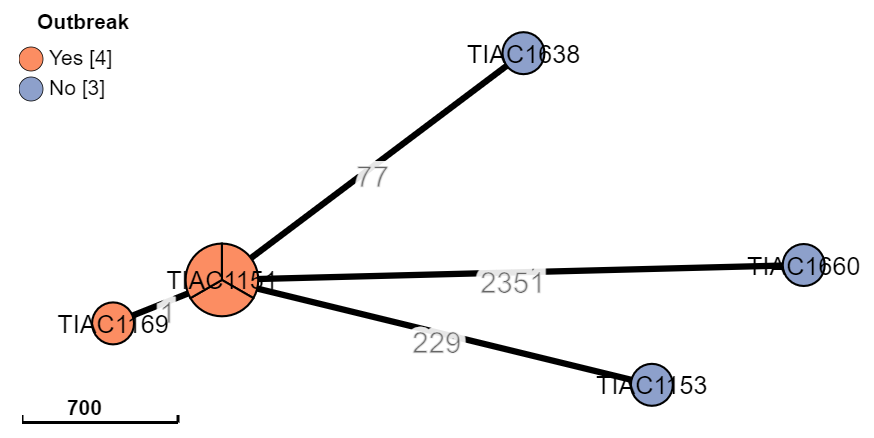


Minimum spanning tree for the *E. coli* isolates constructed based on core genome multi-locus sequence typing (cgMLST). Branch lengths and the scale bar are expressed as number of allelic differences. Branch lengths were logarithmically scaled. The total number of loci in the cgMLST scheme was 2,513. Outbreak isolates are shown in red, unrelated isolates in blue.

## **Figure S28: Core-genome MLST (cgMLST) phylogeny for hybrid assemblies for *L. monocytogenes***


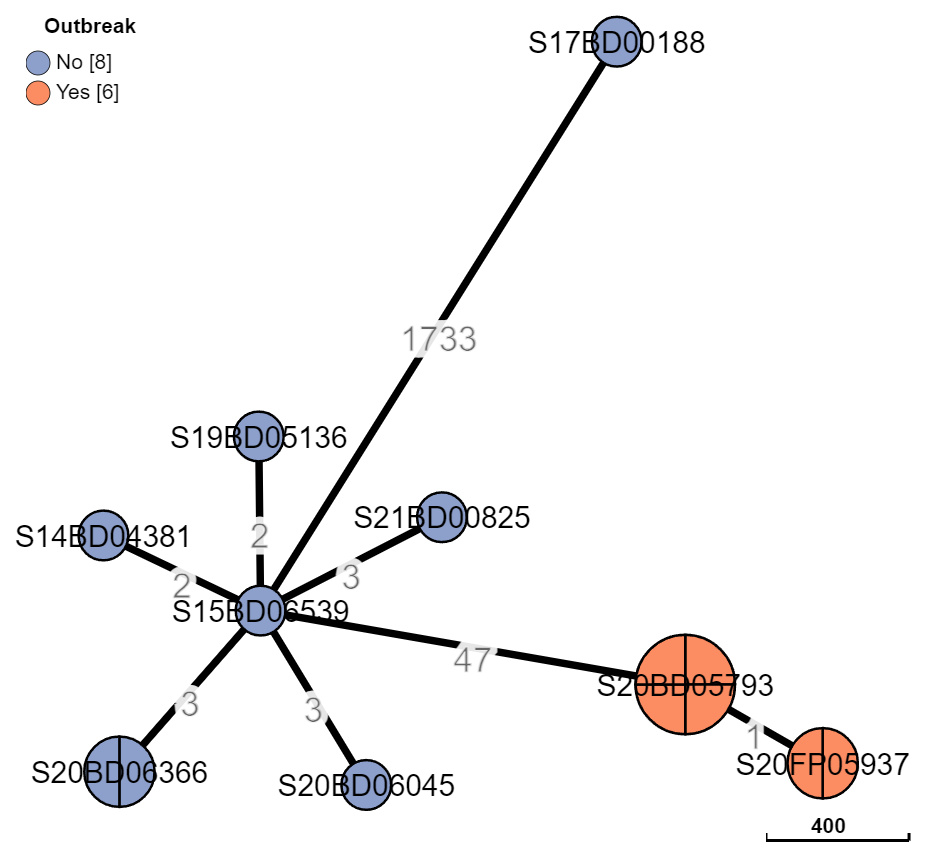


Minimum spanning tree for the *L. monocytogenes* isolates constructed based on core genome multi-locus sequence typing (cgMLST). Branch lengths and the scale bar are expressed as number of allelic differences. Branch lengths were logarithmically scaled. The total number of loci in the cgMLST scheme was 1,748. Outbreak isolates are shown in red, unrelated isolates in blue.

**Supplementary Tables**

## **Table S1: Accession numbers**

| **isolate** | **BioSample** | **R9** | **R10** | **Illumina** |
| --- | --- | --- | --- | --- |
| S14BD04381 | SAMN37324499 | SRR25999225 | SRR25999210 | SRR26086490 |
| S15BD06539 | SAMN37324500 | SRR25999224 | SRR25999209 | SRR26086489 |
| S17BD00188 | SAMN37324501 | SRR25999218 | SRR25999203 | SRR26086483 |
| S19BD05136 | SAMN37324502 | SRR25999217 | SRR25999202 | SRR26086482 |
| S20BD05318 | SAMN37324503 | SRR25999216 | SRR25999201 | SRR26086481 |
| S20BD05447 | SAMN37324504 | SRR25999215 | SRR25999200 | SRR26086480 |
| S20BD05448 | SAMN37324505 | SRR25999214 | SRR25999199 | SRR26086479 |
| S20BD05793 | SAMN37324506 | SRR25999213 | SRR25999198 | SRR26086478 |
| S20BD06045 | SAMN37324507 | SRR25999212 | SRR25999197 | SRR26086477 |
| S20BD06046 | SAMN37324508 | SRR25999211 | SRR25999196 | SRR26086476 |
| S20BD06366 | SAMN37324509 | SRR25999223 | SRR25999208 | SRR26086488 |
| S20BD06374 | SAMN37324510 | SRR25999222 | SRR25999207 | SRR26086487 |
| S20BD06739 | SAMN37324511 | SRR25999221 | SRR25999206 | SRR26086486 |
| S20FP05937 | SAMN37324512 | SRR25999220 | SRR25999205 | SRR26086485 |
| S21BD00825 | SAMN37324513 | SRR25999219 | SRR25999204 | SRR26086484 |
| TIAC1151 | SAMN12871462 | SRR26036477 | SRR26036458 | SRR10201424 |
| TIAC1152 | SAMN12871463 | SRR26036476 | SRR26036457 | SRR10201464 |
| TIAC1153 | SAMN12871464 | SRR26036475 | SRR26036456 | SRR10201455 |
| TIAC1165 | SAMN12871465 | SRR26036474 | SRR26036455 | SRR10201438 |
| TIAC1169 | SAMN12871466 | SRR26036473 | SRR26036454 | SRR10201412 |
| TIAC1638 | SAMN12871467 | SRR26036472 | SRR26036453 | SRR10201405 |
| TIAC1660 | SAMN12871468 | SRR26036471 | SRR26036452 | SRR10201393 |

## **Table S2: Selection mixed datasets *E. coli***

| **Combination** | **Replicate** | **TIAC1153** | **TIAC1151** | **TIAC1169** | **TIAC1165** | **TIAC1152** | **TIAC1638** |
| --- | --- | --- | --- | --- | --- | --- | --- |
| R10 - Illumina | 1 | Illumina | Illumina | R10 | Illumina | R10 | Illumina |
| R10 - Illumina | 2 | R10 | R10 | Illumina | Illumina | R10 | Illumina |
| R10 - Illumina | 3 | Illumina | R10 | Illumina | R10 | Illumina | Illumina |
| R10 - Illumina | 4 | Illumina | R10 | R10 | Illumina | R10 | Illumina |
| R10 - Illumina | 5 | R10 | R10 | R10 | Illumina | Illumina | R10 |
| R10 - Illumina | 6 | R10 | R10 | R10 | R10 | Illumina | Illumina |
| R10 - Illumina | 7 | R10 | R10 | Illumina | R10 | R10 | R10 |
| R10 - Illumina | 8 | R10 | R10 | R10 | R10 | R10 | R10 |
| R10 - Illumina | 9 | Illumina | R10 | R10 | Illumina | R10 | R10 |
| R10 - Illumina | 10 | R10 | R10 | Illumina | Illumina | R10 | R10 |
| R9 - Illumina | 1 | Illumina | R9 | R9 | R9 | Illumina | Illumina |
| R9 - Illumina | 2 | Illumina | Illumina | Illumina | R9 | Illumina | Illumina |
| R9 - Illumina | 3 | Illumina | Illumina | R9 | Illumina | R9 | R9 |
| R9 - Illumina | 4 | R9 | Illumina | Illumina | R9 | Illumina | R9 |
| R9 - Illumina | 5 | Illumina | Illumina | Illumina | Illumina | R9 | R9 |
| R9 - Illumina | 6 | Illumina | Illumina | Illumina | R9 | Illumina | R9 |
| R9 - Illumina | 7 | Illumina | Illumina | Illumina | R9 | Illumina | R9 |
| R9 - Illumina | 8 | Illumina | R9 | R9 | Illumina | Illumina | R9 |
| R9 - Illumina | 9 | R9 | Illumina | Illumina | R9 | Illumina | R9 |
| R9 - Illumina | 10 | R9 | Illumina | Illumina | R9 | R9 | Illumina |
| R9 - R10 | 1 | R9 | R9 | R10 | R9 | R10 | R9 |
| R9 - R10 | 2 | R10 | R10 | R9 | R10 | R9 | R9 |
| R9 - R10 | 3 | R10 | R9 | R10 | R10 | R10 | R10 |
| R9 - R10 | 4 | R9 | R9 | R10 | R9 | R9 | R10 |
| R9 - R10 | 5 | R10 | R10 | R9 | R9 | R9 | R10 |
| R9 - R10 | 6 | R10 | R10 | R9 | R9 | R10 | R10 |
| R9 - R10 | 7 | R9 | R9 | R10 | R9 | R9 | R9 |
| R9 - R10 | 8 | R10 | R10 | R10 | R9 | R10 | R10 |
| R9 - R10 | 9 | R9 | R10 | R9 | R9 | R9 | R9 |
| R9 - R10 | 10 | R9 | R10 | R9 | R9 | R10 | R10 |
| R9 - R10 - Illumina | 1 | R10 | R9 | R9 | R10 | Illumina | R10 |
| R9 - R10 - Illumina | 2 | R9 | R10 | R9 | R10 | Illumina | R9 |
| R9 - R10 - Illumina | 3 | Illumina | R10 | R9 | R10 | Illumina | R9 |
| R9 - R10 - Illumina | 4 | Illumina | Illumina | R10 | Illumina | Illumina | R9 |
| R9 - R10 - Illumina | 5 | R9 | R10 | R10 | R10 | Illumina | Illumina |
| R9 - R10 - Illumina | 6 | Illumina | R9 | R10 | R9 | R10 | Illumina |
| R9 - R10 - Illumina | 7 | R9 | Illumina | R9 | Illumina | R10 | Illumina |
| R9 - R10 - Illumina | 8 | R9 | R10 | R10 | R9 | Illumina | Illumina |
| R9 - R10 - Illumina | 9 | Illumina | R9 | R9 | R9 | R10 | R10 |
| R9 - R10 - Illumina | 10 | R9 | R10 | R9 | R9 | R10 | R9 |

## **Table S3: Selection mixed datasets *L. monocytogenes***

| **Combination** | **Replicate** | **S14BD04381** | **S19BD05136** | **S20BD05318** | **S20BD05447** | **S20BD05793** | **S20BD06045** | **S20BD06046** | **S20BD06366** | **S20BD06374** | **S20BD06739** | **S20FP05937** | **S21BD00825** |
| --- | --- | --- | --- | --- | --- | --- | --- | --- | --- | --- | --- | --- | --- |
| R9 - R10 | 1 | R10 | R9 | R10 | R10 | R9 | R9 | R9 | R9 | R9 | R9 | R9 | R9 |
| R9 - R10 | 2 | R10 | R9 | R10 | R10 | R10 | R10 | R10 | R10 | R9 | R10 | R10 | R10 |
| R9 - R10 | 3 | R10 | R9 | R9 | R9 | R9 | R9 | R10 | R9 | R9 | R9 | R10 | R9 |
| R9 - R10 | 4 | R9 | R10 | R9 | R9 | R10 | R9 | R9 | R9 | R9 | R9 | R9 | R9 |
| R9 - R10 | 5 | R9 | R9 | R9 | R9 | R9 | R9 | R9 | R10 | R10 | R10 | R10 | R10 |
| R9 - R10 | 6 | R9 | R9 | R9 | R10 | R9 | R10 | R10 | R9 | R9 | R10 | R9 | R9 |
| R9 - R10 | 7 | R10 | R10 | R10 | R10 | R10 | R10 | R10 | R10 | R10 | R9 | R10 | R10 |
| R9 - R10 | 8 | R9 | R10 | R10 | R10 | R9 | R9 | R9 | R10 | R10 | R10 | R9 | R9 |
| R9 - R10 | 9 | R10 | R9 | R9 | R10 | R9 | R9 | R9 | R10 | R9 | R10 | R9 | R9 |
| R9 - R10 | 10 | R10 | R10 | R10 | R9 | R10 | R10 | R10 | R10 | R9 | R9 | R10 | R10 |
| R9 - ilmn | 1 | ilmn | R9 | ilmn | R9 | R9 | ilmn | R9 | ilmn | ilmn | ilmn | ilmn | R9 |
| R9 - ilmn | 2 | R9 | R9 | ilmn | ilmn | R9 | R9 | ilmn | R9 | ilmn | ilmn | ilmn | R9 |
| R9 - ilmn | 3 | ilmn | R9 | ilmn | R9 | R9 | R9 | R9 | R9 | R9 | R9 | R9 | R9 |
| R9 - ilmn | 4 | R9 | ilmn | R9 | ilmn | ilmn | R9 | ilmn | R9 | R9 | ilmn | R9 | R9 |
| R9 - ilmn | 5 | ilmn | R9 | R9 | R9 | R9 | R9 | R9 | ilmn | ilmn | R9 | ilmn | ilmn |
| R9 - ilmn | 6 | ilmn | R9 | ilmn | R9 | R9 | R9 | R9 | ilmn | ilmn | R9 | ilmn | R9 |
| R9 - ilmn | 7 | R9 | R9 | ilmn | ilmn | ilmn | ilmn | R9 | R9 | R9 | ilmn | ilmn | ilmn |
| R9 - ilmn | 8 | ilmn | R9 | R9 | R9 | ilmn | ilmn | R9 | ilmn | R9 | ilmn | R9 | ilmn |
| R9 - ilmn | 9 | R9 | R9 | R9 | R9 | ilmn | R9 | R9 | R9 | ilmn | R9 | R9 | ilmn |
| R9 - ilmn | 10 | R9 | R9 | R9 | ilmn | R9 | R9 | R9 | ilmn | R9 | R9 | ilmn | R9 |
| R10 - ilmn | 1 | R10 | ilmn | R10 | R10 | R10 | R10 | ilmn | R10 | R10 | R10 | ilmn | ilmn |
| R10 - ilmn | 2 | ilmn | R10 | ilmn | ilmn | ilmn | R10 | R10 | R10 | ilmn | R10 | R10 | ilmn |
| R10 - ilmn | 3 | R10 | R10 | ilmn | ilmn | R10 | ilmn | R10 | R10 | ilmn | R10 | ilmn | ilmn |
| R10 - ilmn | 4 | ilmn | ilmn | R10 | ilmn | R10 | ilmn | R10 | R10 | R10 | R10 | ilmn | ilmn |
| R10 - ilmn | 5 | R10 | R10 | ilmn | ilmn | ilmn | R10 | ilmn | R10 | R10 | R10 | ilmn | ilmn |
| R10 - ilmn | 6 | ilmn | R10 | R10 | R10 | R10 | R10 | ilmn | ilmn | R10 | ilmn | R10 | ilmn |
| R10 - ilmn | 7 | R10 | ilmn | ilmn | ilmn | ilmn | R10 | R10 | R10 | ilmn | ilmn | R10 | R10 |
| R10 - ilmn | 8 | ilmn | ilmn | R10 | ilmn | ilmn | ilmn | R10 | ilmn | ilmn | R10 | R10 | ilmn |
| R10 - ilmn | 9 | R10 | ilmn | ilmn | ilmn | R10 | R10 | R10 | R10 | R10 | R10 | ilmn | R10 |
| R10 - ilmn | 10 | ilmn | ilmn | ilmn | ilmn | ilmn | ilmn | R10 | R10 | R10 | ilmn | ilmn | R10 |
| R9 - R10 - ilmn | 1 | ilmn | R9 | R10 | R10 | ilmn | ilmn | R10 | R10 | R10 | ilmn | R9 | R10 |
| R9 - R10 - ilmn | 2 | ilmn | ilmn | R10 | ilmn | R9 | R9 | R10 | R9 | R9 | R9 | R10 | ilmn |
| R9 - R10 - ilmn | 3 | ilmn | ilmn | R10 | R9 | R9 | R10 | R9 | ilmn | R10 | R10 | R10 | R9 |
| R9 - R10 - ilmn | 4 | R9 | R9 | R9 | R10 | ilmn | R10 | ilmn | R9 | ilmn | ilmn | ilmn | R10 |
| R9 - R10 - ilmn | 5 | ilmn | R9 | ilmn | ilmn | R10 | R9 | Ilmn | ilmn | R9 | R10 | ilmn | R9 |
| R9 - R10 - ilmn | 6 | ilmn | R10 | R9 | R9 | ilmn | R10 | ilmn | R10 | R9 | R9 | R10 | R10 |
| R9 - R10 - ilmn | 7 | R10 | R9 | R10 | R10 | R10 | R9 | R10 | ilmn | ilmn | R9 | R9 | R10 |
| R9 - R10 - ilmn | 8 | ilmn | R10 | R10 | R10 | ilmn | ilmn | R9 | R10 | R9 | R9 | R10 | R10 |
| R9 - R10 - ilmn | 9 | R9 | R10 | R10 | R9 | ilmn | R10 | ilmn | R10 | ilmn | ilmn | ilmn | ilmn |
| R9 - R10 - ilmn | 10 | ilmn | ilmn | R10 | R9 | R10 | R10 | ilmn | R9 | ilmn | R9 | ilmn | R9 |

Abbreviations: Illumina (ilmn).

## **Table S4: Illumina read statistics**

| **Isolate** | **Species** | **Read pairs** | **Read pairs after trimming** | **Fwd. only surviving** | **Rev. only surviving** | **Orphaned** |
| --- | --- | --- | --- | --- | --- | --- |
| TIAC1151 | *E. coli* | 549,241 | 493,781 | 46,349 | 3,071 | 6,040 |
| TIAC1152 | *E. coli* | 448,903 | 399,607 | 41,423 | 2,471 | 5,402 |
| TIAC1153 | *E. coli* | 635,343 | 578,777 | 46,664 | 3,742 | 6,160 |
| TIAC1165 | *E. coli* | 488,304 | 442,898 | 35,476 | 4,271 | 5,659 |
| TIAC1169 | *E. coli* | 707,090 | 639,548 | 55,721 | 4,296 | 7,525 |
| TIAC1638 | *E. coli* | 501,042 | 462,397 | 24,766 | 8,591 | 5,288 |
| TIAC1660 | *E. coli* | 683,965 | 608,504 | 64,066 | 3,539 | 7,856 |
| S14BD04381 | *L. monocytogenes* | 368,151 | 342,020 | 22,788 | 1,284 | 2,059 |
| S15BD06539 | *L. monocytogenes* | 412,438 | 388,503 | 20,110 | 1,818 | 2,007 |
| S17BD00188 | *L. monocytogenes* | 626,443 | 587,668 | 28,266 | 4,739 | 5,770 |
| S18BD01215 | *L. monocytogenes* | 573,015 | 529,785 | 35,239 | 3,588 | 4,403 |
| S19BD04883 | *L. monocytogenes* | 455,211 | 423,140 | 27,442 | 1,783 | 2,846 |
| S19BD05136 | *L. monocytogenes* | 356,035 | 324,647 | 27,143 | 1,587 | 2,658 |
| S20BD05318 | *L. monocytogenes* | 387,415 | 366,404 | 18,127 | 1,167 | 1,717 |
| S20BD05447 | *L. monocytogenes* | 484,951 | 447,741 | 32,798 | 1,533 | 2,879 |
| S20BD05448 | *L. monocytogenes* | 544,726 | 501,407 | 38,630 | 1,333 | 3,356 |
| S20BD05793 | *L. monocytogenes* | 405,100 | 374,475 | 26,332 | 1,602 | 2,691 |
| S20BD06043 | *L. monocytogenes* | 309,626 | 286,068 | 20,770 | 967 | 1,821 |
| S20BD06045 | *L. monocytogenes* | 279,281 | 258,380 | 18,524 | 815 | 1,562 |
| S20BD06046 | *L. monocytogenes* | 335,419 | 308,776 | 23,530 | 1,096 | 2,017 |
| S20BD06366 | *L. monocytogenes* | 512,209 | 478,550 | 27,531 | 2,837 | 3,291 |
| S20BD06374 | *L. monocytogenes* | 440,855 | 410,095 | 26,174 | 1,870 | 2,716 |
| S20BD06739 | *L. monocytogenes* | 363,194 | 336,513 | 24,101 | 829 | 1,751 |
| S20FP05937 | *L. monocytogenes* | 592,292 | 539,379 | 45,759 | 2,555 | 4,599 |
| S21BD00825 | *L. monocytogenes* | 571,992 | 530,637 | 34,054 | 3,167 | 4,134 |

Abbreviations: forward (fwd), reverse (rev).

## **Table S5: ONT read statistics**

| **Tech** | **Species** | **Mean read length** | **Mean read quality** | **Median read length** | **Median read quality** | **Nb. of reads** | **Read N50** | **Total bases** |
| --- | --- | --- | --- | --- | --- | --- | --- | --- |
| R9 | *L. monocytogenes* | 3,293 | 12.2 | 1,717 | 12.8 | 1,809,723 | 5,822 | 5.96E+09 |
| R9 | STEC | 2,436 | 12.6 | 1,027 | 13.4 | 4,465,213 | 5,529 | 1.088E+10 |
| R10 | *L. monocytogenes* | 1,714 | 16.5 | 932 | 17.0 | 3,876,476 | 3,059 | 6.645E+09 |
| R10 | STEC | 1,559 | 15.2 | 811 | 16.1 | 5,099,405 | 2,805 | 7.951E+09 |

## **Table S6: Read mapping**

| **Species** | **Isolate** | **Technology** | **Mapping rate (%)** | **Median depth** | **Genome coverage (%)** | **Outgroup** |
| --- | --- | --- | --- | --- | --- | --- |
| *E. coli* | TIAC1151 | Illumina | 100.00 | 69 | 99.00 | No |
| *E. coli* | TIAC1152 | Illumina | 100.00 | 57 | 98.28 | No |
| *E. coli* | TIAC1153 | Illumina | 100.00 | 84 | 96.96 | No |
| *E. coli* | TIAC1165 | Illumina | 100.00 | 63 | 98.85 | No |
| *E. coli* | TIAC1169 | Illumina | 100.00 | 93 | 98.43 | No |
| *E. coli* | TIAC1638 | Illumina | 100.00 | 63 | 99.07 | No |
| *E. coli* | TIAC1660 | Illumina | 100.00 | 81 | 80.26 | Yes |
| *E. coli* | TIAC1151 | ONT R10 | 97.66 | 128 | 99.18 | No |
| *E. coli* | TIAC1152 | ONT R10 | 97.48 | 191 | 98.84 | No |
| *E. coli* | TIAC1153 | ONT R10 | 97.03 | 139 | 97.70 | No |
| *E. coli* | TIAC1165 | ONT R10 | 97.33 | 176 | 99.17 | No |
| *E. coli* | TIAC1169 | ONT R10 | 97.45 | 144 | 98.90 | No |
| *E. coli* | TIAC1638 | ONT R10 | 97.21 | 127 | 99.49 | No |
| *E. coli* | TIAC1660 | ONT R10 | 87.40 | 122 | 85.87 | Yes |
| *E. coli* | TIAC1151 | ONT R9 | 97.76 | 86 | 99.81 | No |
| *E. coli* | TIAC1152 | ONT R9 | 97.65 | 177 | 99.88 | No |
| *E. coli* | TIAC1153 | ONT R9 | 97.49 | 167 | 99.95 | No |
| *E. coli* | TIAC1165 | ONT R9 | 97.60 | 154 | 99.83 | No |
| *E. coli* | TIAC1169 | ONT R9 | 97.76 | 166 | 99.86 | No |
| *E. coli* | TIAC1638 | ONT R9 | 97.31 | 268 | 99.89 | No |
| *E. coli* | TIAC1660 | ONT R9 | 88.05 | 418 | 99.47 | Yes |
| *L. monocytogenes* | S14BD04381 | Illumina | 100.00 | 84 | 99.97 | No |
| *L. monocytogenes* | S15BD06539 | Illumina | 100.00 | 90 | 99.97 | No |
| *L. monocytogenes* | S17BD00188 | Illumina | 100.00 | 114 | 91.84 | Yes |
| *L. monocytogenes* | S18BD01215 | Illumina | 100.00 | 117 | 99.96 | No |
| *L. monocytogenes* | S19BD04883 | Illumina | 100.00 | 99 | 99.95 | No |
| *L. monocytogenes* | S19BD05136 | Illumina | 100.00 | 78 | 99.97 | No |
| *L. monocytogenes* | S20BD05318 | Illumina | 100.00 | 90 | 99.97 | No |
| *L. monocytogenes* | S20BD05447 | Illumina | 100.00 | 114 | 99.97 | No |
| *L. monocytogenes* | S20BD05448 | Illumina | 100.00 | 126 | 99.97 | No |
| *L. monocytogenes* | S20BD05793 | Illumina | 100.00 | 96 | 99.94 | No |
| *L. monocytogenes* | S20BD06043 | Illumina | 100.00 | 69 | 99.97 | No |
| *L. monocytogenes* | S20BD06045 | Illumina | 100.00 | 63 | 99.96 | No |
| *L. monocytogenes* | S20BD06046 | Illumina | 100.00 | 75 | 99.96 | No |
| *L. monocytogenes* | S20BD06366 | Illumina | 100.00 | 114 | 99.95 | No |
| *L. monocytogenes* | S20BD06374 | Illumina | 100.00 | 96 | 99.97 | No |
| *L. monocytogenes* | S20BD06739 | Illumina | 100.00 | 87 | 99.97 | No |
| *L. monocytogenes* | S20FP05937 | Illumina | 100.00 | 141 | 99.97 | No |
| *L. monocytogenes* | S21BD00825 | Illumina | 100.00 | 126 | 99.95 | No |
| *L. monocytogenes* | S14BD04381 | ONT R10 | 92.70 | 112 | 99.97 | No |
| *L. monocytogenes* | S15BD06539 | ONT R10 | 92.86 | 173 | 99.98 | No |
| *L. monocytogenes* | S17BD00188 | ONT R10 | 91.91 | 50 | 94.34 | Yes |
| *L. monocytogenes* | S19BD05136 | ONT R10 | 93.00 | 275 | 99.98 | No |
| *L. monocytogenes* | S20BD05318 | ONT R10 | 98.58 | 62 | 99.97 | No |
| *L. monocytogenes* | S20BD05447 | ONT R10 | 98.50 | 115 | 99.97 | No |
| *L. monocytogenes* | S20BD05448 | ONT R10 | 98.53 | 34 | 99.96 | No |
| *L. monocytogenes* | S20BD05793 | ONT R10 | 98.65 | 165 | 99.98 | No |
| *L. monocytogenes* | S20BD06045 | ONT R10 | 93.24 | 56 | 99.96 | No |
| *L. monocytogenes* | S20BD06046 | ONT R10 | 93.62 | 95 | 99.98 | No |
| *L. monocytogenes* | S20BD06366 | ONT R10 | 93.16 | 269 | 99.98 | No |
| *L. monocytogenes* | S20BD06374 | ONT R10 | 93.34 | 32 | 99.96 | No |
| *L. monocytogenes* | S20BD06739 | ONT R10 | 98.52 | 128 | 99.97 | No |
| *L. monocytogenes* | S20FP05937 | ONT R10 | 98.52 | 62 | 99.97 | No |
| *L. monocytogenes* | S21BD00825 | ONT R10 | 93.49 | 58 | 99.97 | No |
| *L. monocytogenes* | S14BD04381 | ONT R9 | 92.72 | 34 | 99.98 | No |
| *L. monocytogenes* | S15BD06539 | ONT R9 | 93.32 | 9 | 99.76 | No |
| *L. monocytogenes* | S17BD00188 | ONT R9 | 92.83 | 152 | 99.30 | Yes |
| *L. monocytogenes* | S19BD05136 | ONT R9 | 93.08 | 175 | 99.98 | No |
| *L. monocytogenes* | S20BD05318 | ONT R9 | 98.51 | 219 | 99.98 | No |
| *L. monocytogenes* | S20BD05447 | ONT R9 | 98.48 | 227 | 99.98 | No |
| *L. monocytogenes* | S20BD05448 | ONT R9 | 98.92 | 18 | 99.96 | No |
| *L. monocytogenes* | S20BD05793 | ONT R9 | 98.82 | 87 | 99.98 | No |
| *L. monocytogenes* | S20BD06045 | ONT R9 | 93.44 | 75 | 99.98 | No |
| *L. monocytogenes* | S20BD06046 | ONT R9 | 93.92 | 23 | 99.96 | No |
| *L. monocytogenes* | S20BD06366 | ONT R9 | 93.65 | 78 | 99.98 | No |
| *L. monocytogenes* | S20BD06374 | ONT R9 | 93.89 | 54 | 99.98 | No |
| *L. monocytogenes* | S20BD06739 | ONT R9 | 98.64 | 106 | 99.98 | No |
| *L. monocytogenes* | S20FP05937 | ONT R9 | 98.67 | 49 | 99.97 | No |
| *L. monocytogenes* | S21BD00825 | ONT R9 | 93.86 | 89 | 99.98 | No |

## **Table S7: SNP calling and filtering**

| **Species** | **Isolate** | **Technology** | **Nb. SNPs** | **Nb. SNPs (filtered)** |
| --- | --- | --- | --- | --- |
| *E. coli* | TIAC1151 | Illumina | 151 | 149 |
| *E. coli* | TIAC1152 | Illumina | 154 | 148 |
| *E. coli* | TIAC1153 | Illumina | 544 | 532 |
| *E. coli* | TIAC1165 | Illumina | 151 | 148 |
| *E. coli* | TIAC1169 | Illumina | 152 | 150 |
| *E. coli* | TIAC1638 | Illumina | 133 | 132 |
| *E. coli* | TIAC1151 | ONT R10 | 153 | 152 |
| *E. coli* | TIAC1152 | ONT R10 | 153 | 153 |
| *E. coli* | TIAC1153 | ONT R10 | 552 | 537 |
| *E. coli* | TIAC1165 | ONT R10 | 153 | 152 |
| *E. coli* | TIAC1169 | ONT R10 | 154 | 153 |
| *E. coli* | TIAC1638 | ONT R10 | 133 | 133 |
| *E. coli* | TIAC1151 | ONT R9 | 167 | 152 |
| *E. coli* | TIAC1152 | ONT R9 | 156 | 153 |
| *E. coli* | TIAC1153 | ONT R9 | 551 | 533 |
| *E. coli* | TIAC1165 | ONT R9 | 160 | 152 |
| *E. coli* | TIAC1169 | ONT R9 | 157 | 154 |
| *E. coli* | TIAC1638 | ONT R9 | 134 | 134 |
| *L. monocytogenes* | S14BD04381 | Illumina | 90 | 89 |
| *L. monocytogenes* | S19BD05136 | Illumina | 92 | 91 |
| *L. monocytogenes* | S20BD05318 | Illumina | 106 | 105 |
| *L. monocytogenes* | S20BD05447 | Illumina | 104 | 103 |
| *L. monocytogenes* | S20BD05793 | Illumina | 106 | 105 |
| *L. monocytogenes* | S20BD06045 | Illumina | 93 | 90 |
| *L. monocytogenes* | S20BD06046 | Illumina | 94 | 91 |
| *L. monocytogenes* | S20BD06366 | Illumina | 94 | 93 |
| *L. monocytogenes* | S20BD06374 | Illumina | 95 | 91 |
| *L. monocytogenes* | S20BD06739 | Illumina | 107 | 107 |
| *L. monocytogenes* | S20FP05937 | Illumina | 106 | 104 |
| *L. monocytogenes* | S21BD00825 | Illumina | 93 | 91 |
| *L. monocytogenes* | S14BD04381 | ONT R10 | 89 | 89 |
| *L. monocytogenes* | S19BD05136 | ONT R10 | 91 | 91 |
| *L. monocytogenes* | S20BD05318 | ONT R10 | 105 | 105 |
| *L. monocytogenes* | S20BD05447 | ONT R10 | 104 | 104 |
| *L. monocytogenes* | S20BD05793 | ONT R10 | 105 | 105 |
| *L. monocytogenes* | S20BD06045 | ONT R10 | 95 | 92 |
| *L. monocytogenes* | S20BD06046 | ONT R10 | 91 | 91 |
| *L. monocytogenes* | S20BD06366 | ONT R10 | 93 | 93 |
| *L. monocytogenes* | S20BD06374 | ONT R10 | 94 | 92 |
| *L. monocytogenes* | S20BD06739 | ONT R10 | 106 | 106 |
| *L. monocytogenes* | S20FP05937 | ONT R10 | 105 | 105 |
| *L. monocytogenes* | S21BD00825 | ONT R10 | 91 | 91 |
| *L. monocytogenes* | S14BD04381 | ONT R9 | 104 | 87 |
| *L. monocytogenes* | S19BD05136 | ONT R9 | 99 | 90 |
| *L. monocytogenes* | S20BD05318 | ONT R9 | 108 | 105 |
| *L. monocytogenes* | S20BD05447 | ONT R9 | 110 | 103 |
| *L. monocytogenes* | S20BD05793 | ONT R9 | 119 | 105 |
| *L. monocytogenes* | S20BD06045 | ONT R9 | 102 | 90 |
| *L. monocytogenes* | S20BD06046 | ONT R9 | 115 | 89 |
| *L. monocytogenes* | S20BD06366 | ONT R9 | 108 | 93 |
| *L. monocytogenes* | S20BD06374 | ONT R9 | 103 | 92 |
| *L. monocytogenes* | S20BD06739 | ONT R9 | 114 | 103 |
| *L. monocytogenes* | S20FP05937 | ONT R9 | 120 | 104 |
| *L. monocytogenes* | S21BD00825 | ONT R9 | 108 | 90 |
